# Supplementary material for: High reactivity of deep biota under anthropogenic CO2 injection into basalt
Source: Nat Commun. 2017 Oct 20;8:1063. doi: 10.1038/s41467-017-01288-8 (PMC5648843; doi:10.1038/s41467-017-01288-8)
Supplement: Supplementary file 1 — Supplementary Information [file 41467_2017_1288_MOESM1_ESM.pdf]

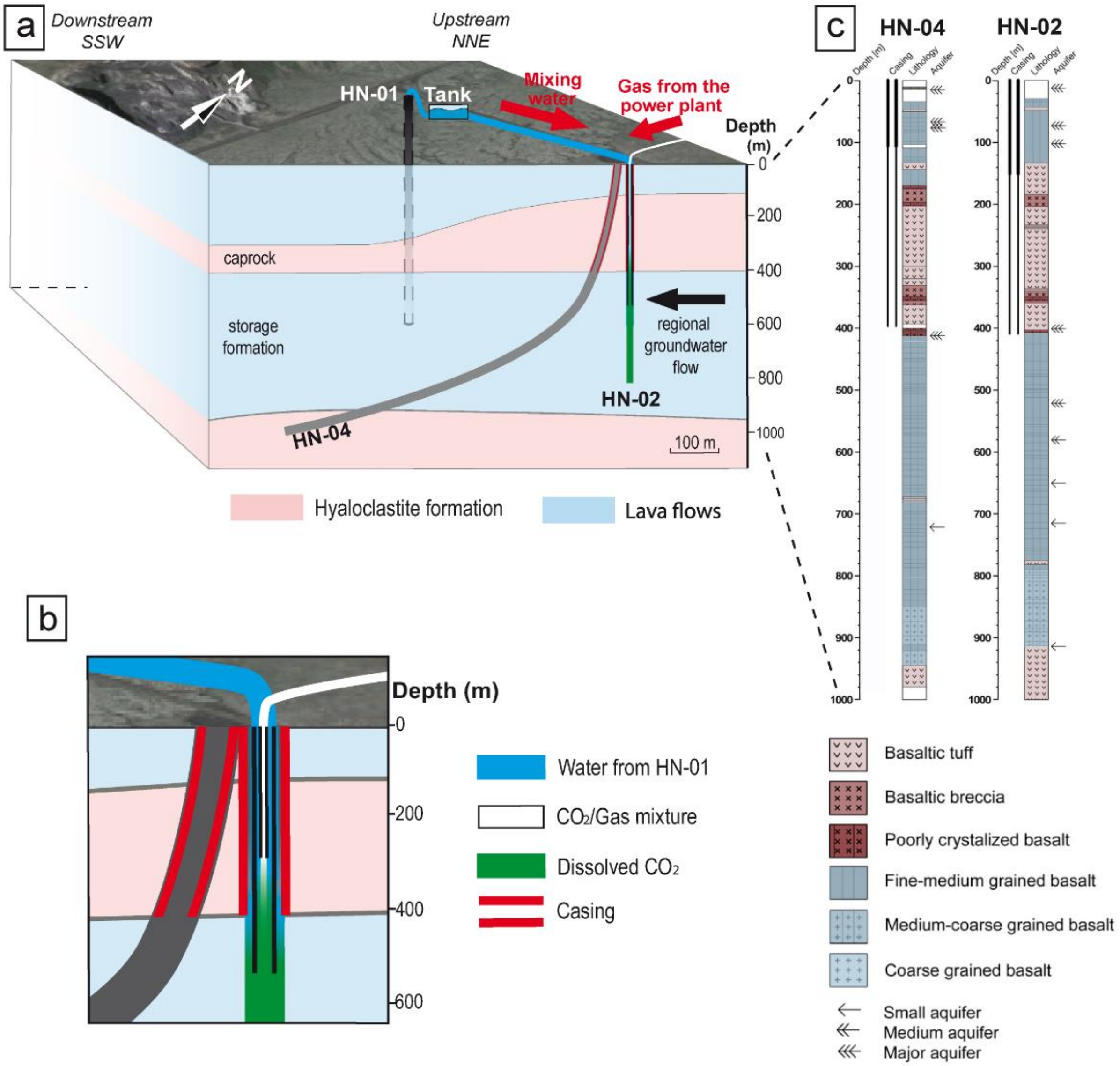

Supplementary Figure 1. Schematic representation of the CarbFix1 injection site (64°02'14''N 21°24'08''W, SW Iceland) located ca. 3 km SW of the Hellisheidi geothermal power plant in the Hengill volcanic area (adapted from[1,2]). Here, CO_2-charged waters (i.e. pure commercial CO_2 and gas mixture produced by the plant) were injected in 2012 with reactive and non-reactive tracers (including ^{14}C, sulfurhexafluoride SF_6, and trifluoromethyl sulfur pentafluoride SF_5CF_3) in the framework of a fully integrated project (https://www.or.is/en/projects/carbfix; Supplementary Table 1). Some of those tracers along with Na-fluorescein (Na-Flu) were previously injected for hydrogeological characterizations (Supplementary Table 1; Supplementary Fig. 2). The CarbFix aim was to assess the feasibility of *in situ* mineral storage in basalts, a long-lasting, thermodynamically stable and environmentally benign solution to reduce CO_2 emissions in the atmosphere[1-4]. By harnessing geothermal steam from the Hengill volcanic system in order to produce electricity and thermal energy, the power plant annually emits, as by-products, 40,000 and 16,000 tons of CO_2 and H_2S of volcanic origin. Before being injected, the geothermal gas coming from the condensers of the power plant is captured and purified by sequential extraction at the gas-separation station. First, a scrubber washes CO_2 and H_2S out from less soluble gas species (H_2, N_2, Ar and CH_4). A deaerator then removes CO_2 and H_2S from the washing water. Next, these latter are separated in a distillation column. This results in a gas mixture composed of 75% CO_2, 24.2% H_2S, 0.8% H_2. For the

Carbfix1 site, the mixture was transported, as high pressurized gas, in a 3-km pipeline (white line in **a** and **b**) to well HN-02 where it was injected with fresh 25°C groundwater (**b**), initially pumped from the upstream well HN-01 at 1300 m depth and transported in a 1 km pipeline (blue line in **a** and **b**). In injection well HN-02, the static water level was at about 100 m depth. For gas-injection, groundwater from HN-01 were pumped down and equilibrated at 300 m depth with respectively ∼25 bar pressure of the CO_2 gas or ∼14 bar pressure of the CO_2–H_2S–H_2 mixture[3,5]. This resulted in a single phase fluid of pH 3.2-4.0 that penetrated the 400-800 m targeted basaltic storage formation, sealed off from atmospheric CO_2, with the main aquifer being located at ∼500 m depth[1]. In this lava flow sequence, initial groundwater pH was comprised between 8.4 and 9.8 and temperature ranged from 20 to 50°C following a near-linear gradient of $80°C·km^{-1}$. CO_2-plume evolution was monitored thanks to a large network of 9 shallow and deep monitoring wells drilled down through the storage formation and regularly positioned along the regional groundwater flow (direction indicated by black arrow on panel **a**) carrying the injected carbonated waters downstream through the bedrock (Supplementary Fig. 15). The intruded formation consisted of low-permeability glassy hyaloclastic basalt acting as caprock (in pink on **a** and **b**) and fine-to-medium grained crystalline basaltic lavas (in blue on **a** and **b**) of olivine tholeiitic composition (~45 to 49% SiO_2) and low-alteration state. Initial rock was composed of plagioclase, pyroxene, olivine and basaltic glass, with carbonates, clay minerals, simple oxides and hydroxides and Ca-zeolites as common alteration minerals. Extensive description of the aquifer chemistry can be found in Alfredsson et al., 2013[1]. The present study focused on the deviated monitoring well HN-04 shown in **a**, which was the closest from the injection well HN-02. Lithology of the rocks as a function of depth in HN-04 monitoring-well and HN-02 injection-well along with casing configuration and major aquifer location are detailed in **c** (modified from Alfredsson et al., 2013[1]). The main permeable sections in HN-02 and HN-04 were located close to 500 and 400 m depth, respectively. Slug-type tracer tests (Supplementary Table 1) and subsequent modeling constrained with field data showed the storage formation to consist of a large volume of relatively homogeneous porous media. Associated hydrological properties were lateral and vertical intrinsic permeabilities of 300 and 1700·mD, respectively and effective matrix porosity of 8.5%[2]. When the primary rock came in contact with the injected weakly-acidic carbonated fluid, dissolution reactions occurred, leaching divalent cations out of the rock matrix (Supplementary Fig. 2 and Supplementary Table 3)[6]. Cations were then expected to react with the dissolved CO_2 and to form carbonates.

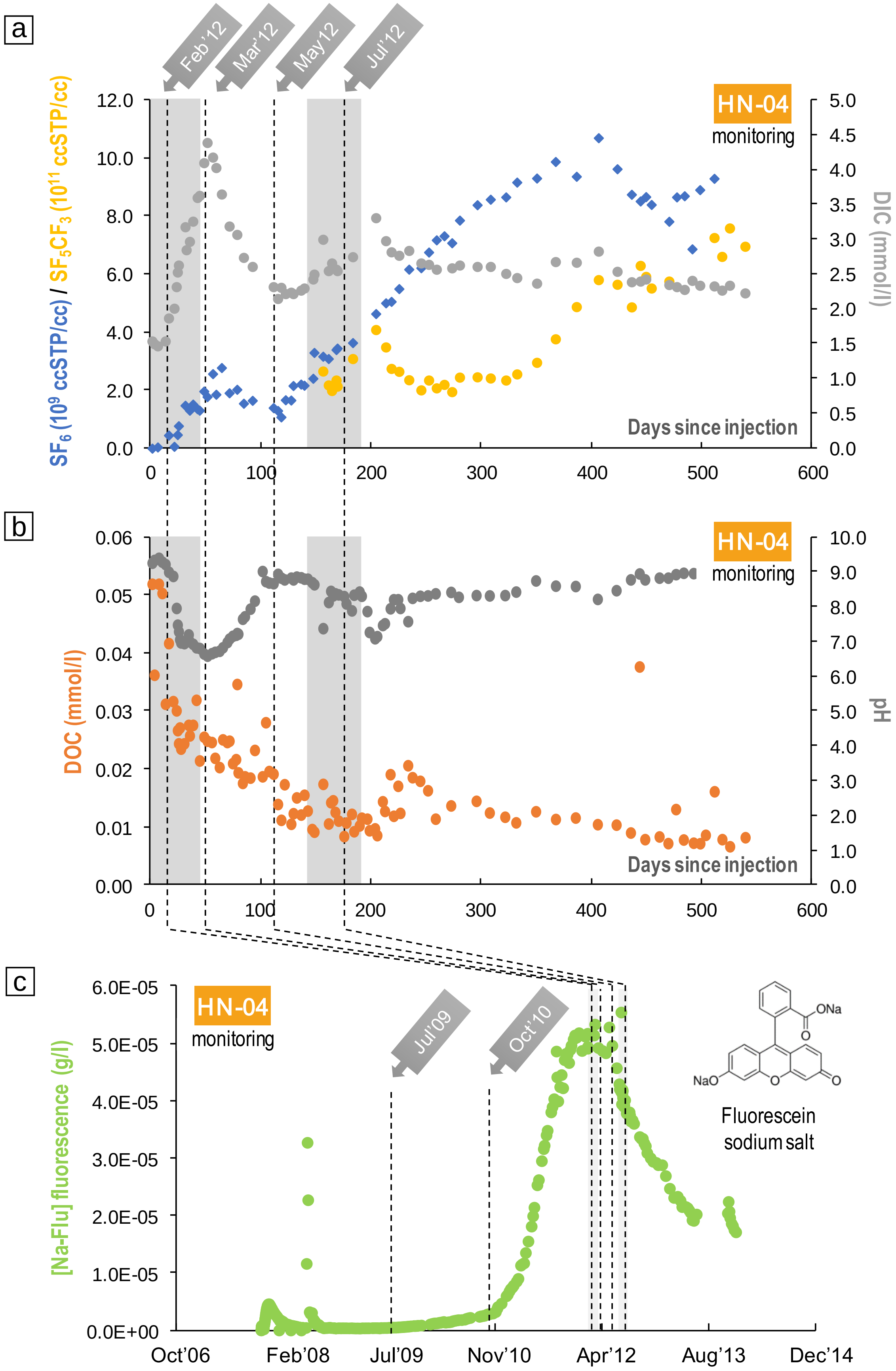
a
Feb'12
Mar'12
May12
Jul'12
HN-04
monitoring
Days since injection
SF6 (10⁹ ccSTP/cc) / SF5CF3 (10¹¹ ccSTP/cc)
DIC (mmol/l)
b
HN-04
monitoring
Days since injection
DOC (mmol/l)
pH
c
HN-04
monitoring
Jul'09
Oct'10
[Na-Flu] fluorescence (g/l)
Oct'06
Feb'08
Jul'09
Nov'10
Apr'12
Aug'13
Dec'14
ONa
NaO
Fluorescein sodium salt

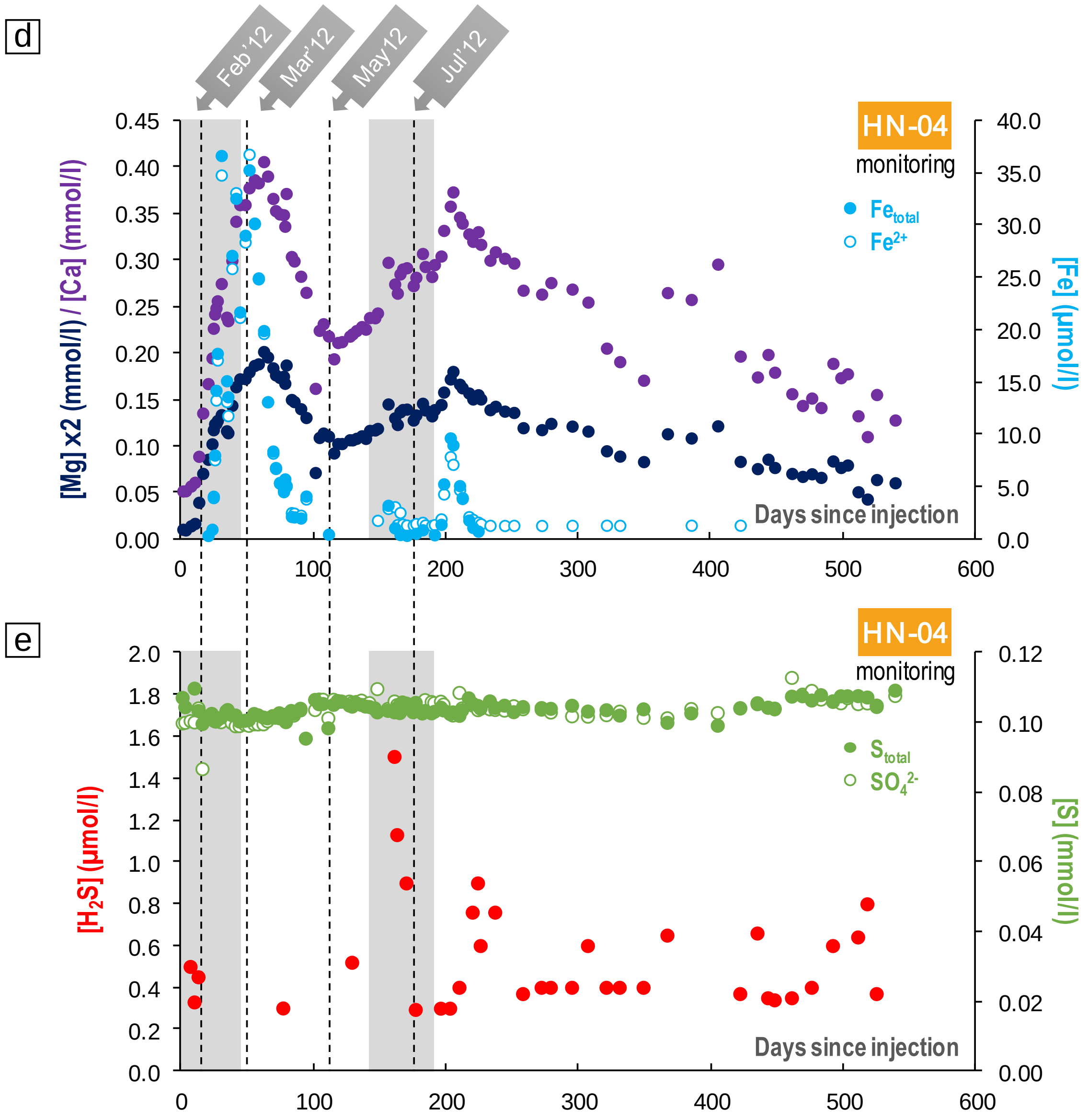

Supplementary Figure 2. Temporal evolution in HN-04 monitoring well of the dissolved inorganic carbon species and non-reactive tracers used for gas injection monitoring and former hydrological characterizations (see Supplementary Table 1 for detailed operations). **(a)** dissolved inorganic carbon (DIC) and non-reactive sulfurhexafluoride (SF_6) and trifluoromethyl sulfur pentafluoride (SF_5CF_3) concentrations measured by Matter et al. (2014) from groundwater pumped in HN-04 monitoring well in the course of the years 2012 and 2013[7]. SF_6 and SF_5CF_3 reflected the expected timing for the plume arrivals associated with the pure CO_2 injection and the geothermal gas mixture, respectively (Supplementary Table 1). For both, the first hump (respectively at ~60 and 200 days since injection) represented the contribution of a fast fracture channeling (~$1.4{\cdot}10^{-5}$ to $2.4{\cdot}10^{-5}$ $m{\cdot}s^{-1}$) through which about 3% of the injected mass flowed while the largest fraction was transported more slowly (~$2{\cdot}10^{-6}$ to $4{\cdot}10^{-6}$ $m{\cdot}s^{-1}$) by matrix flow[2] and was only evidenced by SF_6 measurements after one year[7]. Basalt formations are indeed a dual permeability geological medium wherein large fractures and rubble zones result in high formation permeabilities while the bulk of the basalt matrix has low permeability hence leading to sequential arrivals of the plume. Whereas DIC and SF_6 concentrations presented a similar evolution up to early May 2012 (May12), with DIC values in well HN-04 being maximal in March 2012 (Mar'12), the

trend discrepancies then reflected continuous arrival of the plume while DIC concentrations were significantly lower in the HN-04 groundwater compared to SF_6 values, hence attesting that most of the injected CO_2 had been trapped upstream at depth[7]. The two injection periods are reported with light gray rectangles. Sampling periods for microbiological characterizations are reported as vertical dashed lines. Oct'10, Feb'12 and Jul'12 stand for October 2010, February 2012 and July 2012, respectively. Note that they only encompass the arrivals of the CO_2 fractions that have circulated rapidly through the fracture channeling with little time to react within the aquifer prior to their detection in HN-04 monitoring well[7]. Although representing a small portion of the injected gas, they correspond to the largest unreacted CO_2 fraction that has reached HN-04, as attested by the DIC evolution curve, and hence that allow to illustrate what can be the reactivity of the aquifer microbial inhabitants facing CO_2 enriched groundwater. (**b**) pH and dissolved organic carbon (DOC) concentrations measured by Snæbjörnsdóttir et al. (2017) in groundwater pumped from HN-04 monitoring well in the course of the years 2012 and 2013[6]. (**c**) Long term monitoring of Na-fluorescein (Na-Flu) based on absorbance measurements carried out by the Iceland Geosurvey-ISOR. Those were performed in the framework of former slug-type tracer tests aiming at characterizing the aquifer hydrological properties before gas injections at the Carbfix1 storage site[2,8]; for this purpose, 0.5 kg[8] and 50.0 kg[2] slugs of organic fluorophore were respectively injected in well HN-02 (Supplementary Fig. 1 and Supplementary Table 1) on November, 2007 and June, 2008. As stated previously for panel (**a**), the two small breakthrough curves reflect contribution of fracture channeling while the large hump represents the main fraction transported by matrix flow. The presented chemical structure of Na-fluorescein shows that this molecule is composed of two phenols linked to a pyran ring itself bonded to a benzoic acid. (**d, e**) temporal evolution of Mg, Ca, Fe_{total} and Fe^{2+} concentrations (**d**) and S_{total}, SO_4^{2-} and H_2S concentrations (**e**) measured by Snæbjörnsdóttir et al. (2017) in HN-04 groundwater during the monitoring period[6].

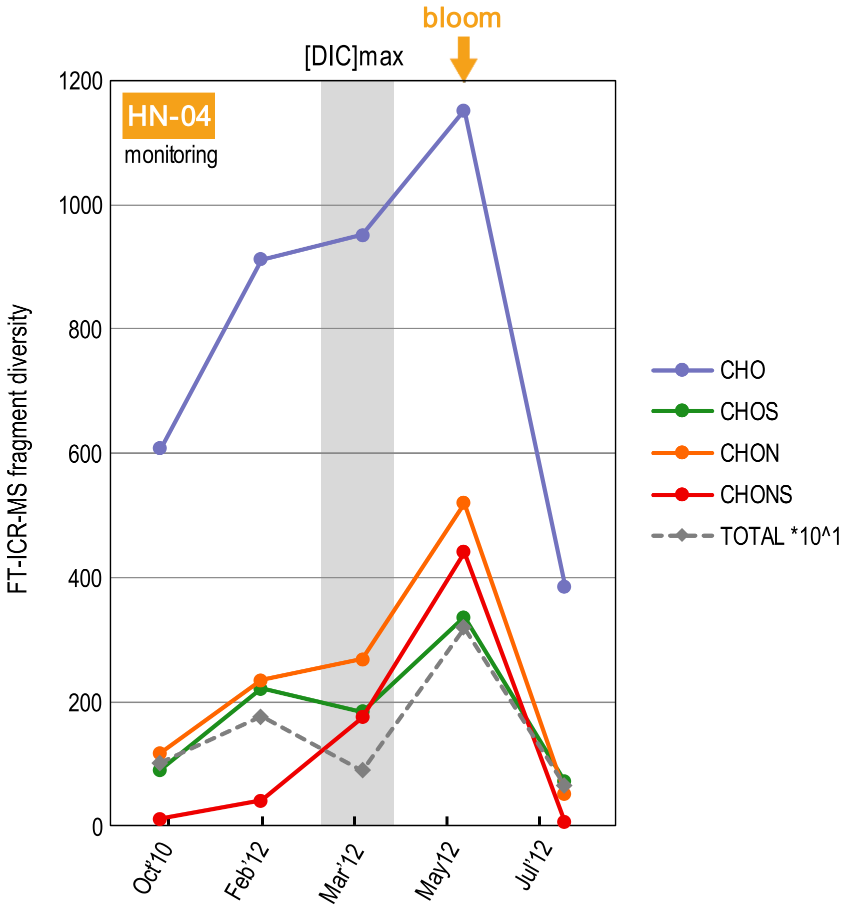

Supplementary Figure 3. Organic fragments detected by electrospray ionization (ESI) Fourier transform-ion cyclotron resonance-mass spectrometry (FT-ICR-MS). It shows concomitantly with the increase in dissolved inorganic carbon (DIC; Supplementary Fig. 2a), an increase of the diversity of dissolved organic compounds, expressed as CHO, CHOS, CHON, CHONS molecular series, in HN-04 groundwater, with maximal values detected in May 2012 (i.e. during the bacterial bloom; Fig. 1e).

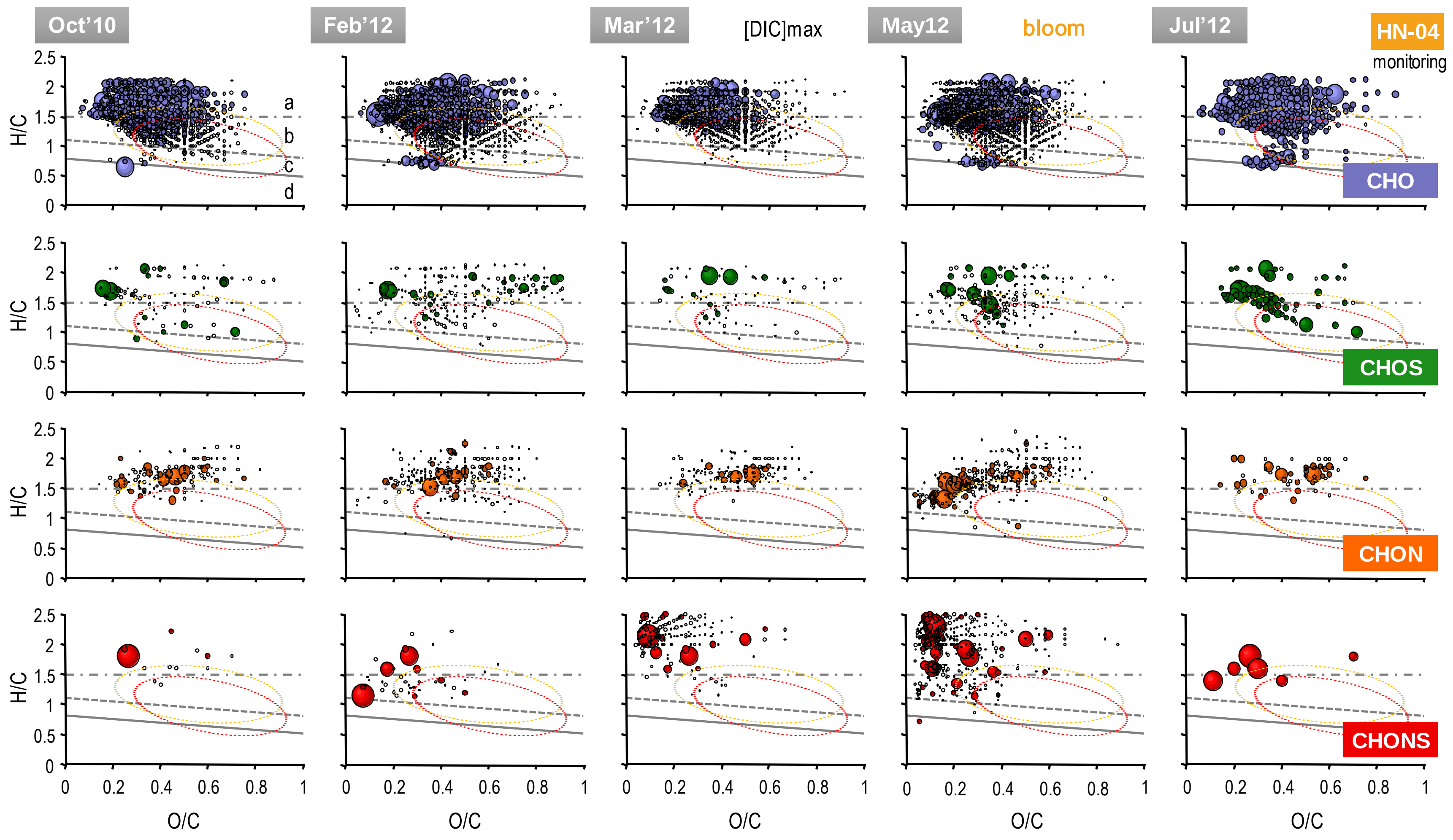

Supplementary Figure 4. Molecular signature of the organic matter present during the monitoring period in HN-04 groundwater and obtained by electrospray ionization (ESI) Fourier transform ion cyclotron resonance/mass spectrometry (FT-ICR-MS). Plots correspond to van Krevelen diagram displaying H/C values as a function of O/C. Circle sizes reflect relative abundances. Grey lines delineate domains for aliphatics (**a**), highly unsaturated and phenolic compounds (**b**) polyphenol (**c**) and polycyclic aromatics (**d**)[9]. Orange and red ellipses respectively

delineate the typical distribution of fresh and aged fulvic acids as found in the soil-derived dissolved organic carbon (DOC) pool of groundwater[10]. CHO distributions in the Hellisheidi groundwater showed in February, May and July'12 an increase in the polycyclic aromatic fraction that may include Na-Fluorescein (Supplementary Fig. 2c) whereas soil-derived DOC is more centered around the (poly)phenolic region even after progressive deoxygenation and hydrogenation associated with aging. Accordingly, the distribution observed for the Hellisheidi groundwater is more indicative of biological oxidization (hydroxylation, carboxylation) and hydrogenation of aliphatic and polyaromatic compounds forming more polar and oxygenated compounds[11,12]. Although C_xH_y compounds are not ionized by using electrospray, their hydroxylated and carboxylated degradation products are in that respect well evidenced in the CHO, CHOS, CHON, CHONS molecular series at $H/C<1$. The increased abundance of nitrogen-bearing compounds in May 2012 (i.e. during the bacterial bloom; Fig. 1e) can be related to an abundance of peptides in the screened mass domain, and is hence also indicative of an increased biological activity.

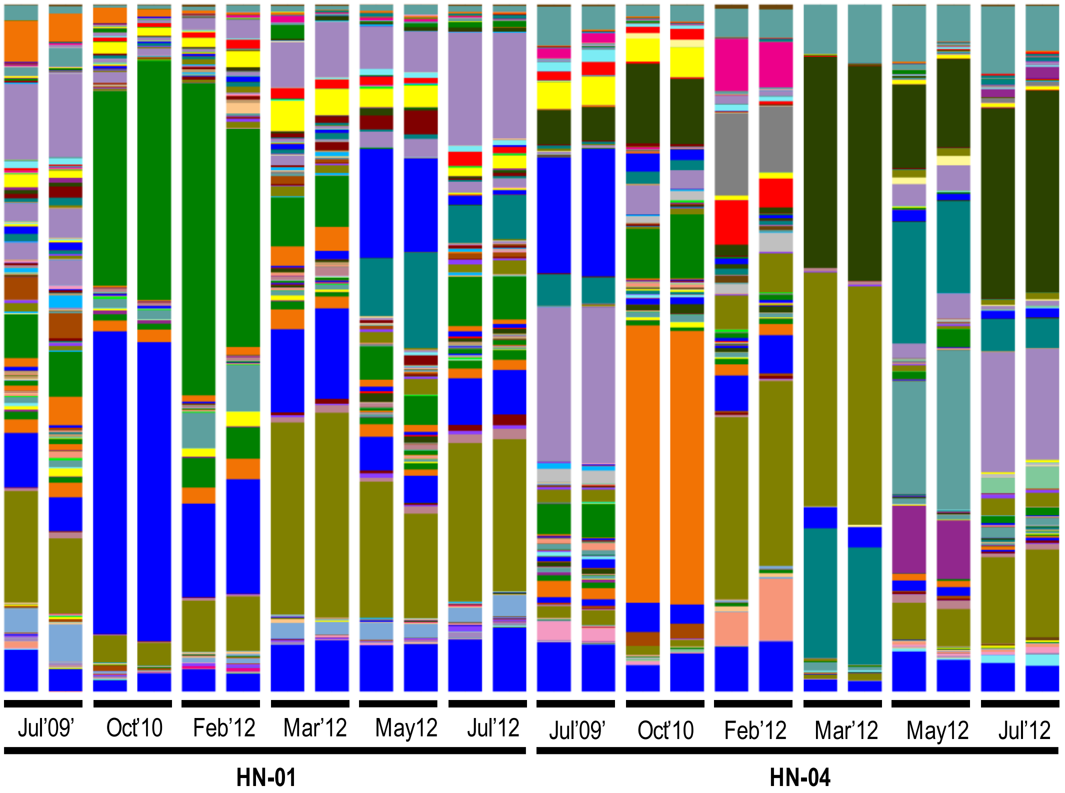

Archaea; Thaumarchaeota; Marine Group I; Candidatus *Nitrosoarchaeum*
Bacteria
Bacteria; Acidobacteria
Bacteria; Acidobacteria; Acidobacteria
Bacteria; Acidobacteria; Acidobacteria; AMGG11
Bacteria; Acidobacteria; Acidobacteria; BPC102
Bacteria; Acidobacteria; Acidobacteria; Candidatus *Chloracidobacterium*
Bacteria; Acidobacteria; Acidobacteria; DA023
Bacteria; Acidobacteria; Acidobacteria; DA052
Bacteria; Acidobacteria; Acidobacteria; JH-WHS99
Bacteria; Acidobacteria; Acidobacteria; SEG-08-550
Bacteria; Acidobacteria; Holophagae; Holophagales; Holophagaceae
Bacteria; Acidobacteria; Holophagae; iii1-8
Bacteria; Actinobacteria
Bacteria; Actinobacteria; Acidimicrobiia; Acidimicrobiales
Bacteria; Actinobacteria; Acidimicrobiia; Acidimicrobiales; Acidimicrobiaceae
Bacteria; Actinobacteria; Acidimicrobiia; Acidimicrobiales; Acidimicrobiaceae; CL500-29 marine group
Bacteria; Actinobacteria; Acidimicrobiia; Acidimicrobiales; TM214
Bacteria; Actinobacteria; Actinobacteria
Bacteria; Actinobacteria; Actinobacteria; Corynebacteriales
Bacteria; Actinobacteria; Actinobacteria; Corynebacteriales; Corynebacteriaceae; *Corynebacterium*
Bacteria; Actinobacteria; Actinobacteria; Corynebacteriales; Corynebacteriaceae
Bacteria; Actinobacteria; Actinobacteria; Corynebacteriales; Mycobacteriaceae; *Mycobacterium*
Bacteria; Actinobacteria; Actinobacteria; Frankiales; Sporichthyaceae
Bacteria; Actinobacteria; Actinobacteria; Frankiales; Sporichthyaceae; Candidatus *Planktophila*
Bacteria; Actinobacteria; Actinobacteria; Micrococcales; Brevibacteriaceae; *Brevibacterium*
Bacteria; Actinobacteria; Actinobacteria; Micrococcales; Intrasporangiaceae
Bacteria; Actinobacteria; Actinobacteria; Micrococcales; Microbacteriaceae
Bacteria; Actinobacteria; Actinobacteria; Micrococcales; Microbacteriaceae; *Microbacterium*
Bacteria; Actinobacteria; Actinobacteria; Micrococcales; Micrococcaceae; *Kocuria*
Bacteria; Actinobacteria; Actinobacteria; Micrococcales; Micrococcaceae; *Micrococcus*
Bacteria; Actinobacteria; Actinobacteria; Micromonosporales; Micromonosporaceae
Bacteria; Actinobacteria; Actinobacteria; Micromonosporales; Micromonosporaceae; *Actinoplanes*
Bacteria; Actinobacteria; Actinobacteria; Propionibacteriales; Nocardioidaceae
Bacteria; Actinobacteria; Actinobacteria; Propionibacteriales; Nocardioidaceae; *Nocardioides*
Bacteria; Actinobacteria; Actinobacteria; Propionibacteriales; Propionibacteriaceae; *Propionibacterium*
Bacteria; Actinobacteria; Coriobacteriia; Coriobacteriales; Coriobacteriaceae
Bacteria; Actinobacteria; MB-A2-108
Bacteria; Actinobacteria; OPB41
Bacteria; Actinobacteria; Thermoleophilia; Gaiellales; uncultured
Bacteria; Armatimonadetes
Bacteria; Armatimonadetes
Bacteria; BD1-5
Bacteria; Bacteroidetes
Bacteria; Bacteroidetes; Bacteroidia; Bacteroidales; ML635J-40 aquatic group
Bacteria; Bacteroidetes; Bacteroidia; Bacteroidales; Marinilabiaceae; *Alkaliflexus*
Bacteria; Bacteroidetes; Bacteroidia; Bacteroidales; Porphyromonadaceae; *Dysgonomonas*
Bacteria; Bacteroidetes; Bacteroidia; Bacteroidales; Porphyromonadaceae; *Paludibacter*
Bacteria; Bacteroidetes; Bacteroidia; Bacteroidales; Prevotellaceae; *Prevotella*
Bacteria; Bacteroidetes; Cytophagia; Cytophagales; Cyclobacteriaceae; *Algoriphagus*
Bacteria; Bacteroidetes; Cytophagia; Cytophagales; Cytophagaceae; *Arcicella*
Bacteria; Bacteroidetes; Cytophagia; Cytophagales; Cytophagaceae; *Flexibacter*
Bacteria; Bacteroidetes; Cytophagia; Cytophagales; Cytophagaceae; *Larkinella*
Bacteria; Bacteroidetes; Cytophagia; Cytophagales; Cytophagaceae; *Spirosoma*
Bacteria; Bacteroidetes; Cytophagia; Cytophagales; Cytophagaceae; *Sporocytophaga*
Bacteria; Bacteroidetes; Flavobacteria; Flavobacteriales
Bacteria; Bacteroidetes; Flavobacteria; Flavobacteriales; Cryomorphaceae; *Fluviicola*
Bacteria; Bacteroidetes; Flavobacteria; Flavobacteriales; Flavobacteriaceae
Bacteria; Bacteroidetes; Flavobacteria; Flavobacteriales; Flavobacteriaceae; *Chryseobacterium*
Bacteria; Bacteroidetes; Flavobacteria; Flavobacteriales; Flavobacteriaceae; *Cloacibacterium*
Bacteria; Bacteroidetes; Flavobacteria; Flavobacteriales; Flavobacteriaceae; *Psychroserpens*
Bacteria; Bacteroidetes; SB-1
Bacteria; Bacteroidetes; Sphingobacteriia; Sphingobacteriales; AKYH767
Bacteria; Bacteroidetes; Sphingobacteriia; Sphingobacteriales; Chitinophagaceae
Bacteria; Bacteroidetes; Sphingobacteriia; Sphingobacteriales; Chitinophagaceae; *Chitinophaga*
Bacteria; Bacteroidetes; Sphingobacteriia; Sphingobacteriales; Chitinophagaceae; *Ferruginibacter*
Bacteria; Bacteroidetes; Sphingobacteriia; Sphingobacteriales; Chitinophagaceae; *Hydrotalea*
Bacteria; Bacteroidetes; Sphingobacteriia; Sphingobacteriales; Chitinophagaceae; *Lacibacter*
Bacteria; Bacteroidetes; Sphingobacteriia; Sphingobacteriales; Chitinophagaceae
Bacteria; Bacteroidetes; Sphingobacteriia; Sphingobacteriales; KD3-93
Bacteria; Bacteroidetes; Sphingobacteriia; Sphingobacteriales; NS11-12 marine group
Bacteria; Bacteroidetes; Sphingobacteriia; Sphingobacteriales; PHOS-HE51
Bacteria; Bacteroidetes; Sphingobacteriia; Sphingobacteriales; Saprospiraceae
Bacteria; Bacteroidetes; Sphingobacteriia; Sphingobacteriales; Sphingobacteriaceae; *Mucilaginibacter*
Bacteria; Bacteroidetes; Sphingobacteriia; Sphingobacteriales; env.OPS 17
Bacteria; Bacteroidetes; VC2.1 Bac22
Bacteria; Bacteroidetes; WCHB1-32
Bacteria; Bacteroidetes; vadinHA17
Bacteria; Candidate division BRC1
Bacteria; Candidate division KB1
Bacteria; Candidate division OD1
Bacteria; Candidate division OP11
Bacteria; Candidate division OP3
Bacteria; Candidate division SR1
Bacteria; Candidate division TM7
Bacteria; Candidate division WS3
Bacteria; Chlorobi; Chlorobia; Chlorobiales; OPB56
Bacteria; Chlorobi; Chlorobia; Chlorobiales; SJA-28
Bacteria; Chlorobi; Ignavibacteria; Ignavibacteriales
Bacteria; Chlorobi; Ignavibacteria; Ignavibacteriales; BSV26
Bacteria; Chlorobi; Ignavibacteria; Ignavibacteriales; SM1H02
Bacteria; Chlorobi; Ignavibacteria; Ignavibacteriales; SR-FBR-L83
Bacteria; Chloroflexi
Bacteria; Chloroflexi; Anaerolineae; Anaerolineales; Anaerolineaceae
Bacteria; Chloroflexi; Anaerolineae; Anaerolineales; Anaerolineaceae; *Bellilinea*
Bacteria; Chloroflexi; Anaerolineae; Anaerolineales; Anaerolineaceae
Bacteria; Chloroflexi; Caldilineae; Caldilineales; Caldilineaceae; *Caldilinea*
Bacteria; Chloroflexi; Caldilineae; Caldilineales; Caldilineaceae
Bacteria; Chloroflexi; GIF9
Bacteria; Chloroflexi; JG30-KF-CM66
Bacteria; Chloroflexi; KD4-96
Bacteria; Chloroflexi; MSBL5
Bacteria; Chloroflexi; P2-11E
Bacteria; Chloroflexi; S085
Bacteria; Chloroflexi; SAR202 clade
Bacteria; Chloroflexi; SHA-26
Bacteria; Chloroflexi; TK10
Bacteria; Chloroflexi; Thermomicrobia; JG30-KF-CM45
Bacteria; Cyanobacteria; Chloroplast; Chloroplast; Chloroplast; Chloroplast
Bacteria; Cyanobacteria; Cyanobacteria; SubsectionIII; FamilyI; *Leptolyngbya*
Bacteria; Cyanobacteria; Cyanobacteria; SubsectionIII; FamilyI; *Phormidium*
Bacteria; Cyanobacteria; MLE1-12
Bacteria; Cyanobacteria; SHA-109
Bacteria; Deferribacteres; Deferribacteres; Deferribacterales; PAUC34f
Bacteria; Deinococcus-Thermus; Deinococci; Thermales; Thermaceae; *Meiothermus*
Bacteria; Deinococcus-Thermus; Deinococci; Thermales; Thermaceae; *Thermus*
Bacteria; Elusimicrobia; Elusimicrobia
Bacteria; Elusimicrobia; Elusimicrobia; 12-31
Bacteria; Elusimicrobia; Elusimicrobia; Lineage IIc
Bacteria; Elusimicrobia; Elusimicrobia; Lineage IV
Bacteria; Fibrobacteres; Fibrobacteria; Fibrobacterales
Bacteria; Fibrobacteres; Fibrobacteria; Fibrobacterales; FD035
Bacteria; Firmicutes
Bacteria; Firmicutes; Bacilli; Bacillales
Bacteria; Firmicutes; Bacilli; Bacillales; Alicyclobacillaceae
Bacteria; Firmicutes; Bacilli; Bacillales; Alicyclobacillaceae; *Tumebacillus*
Bacteria; Firmicutes; Bacilli; Bacillales; Bacillaceae; *Anaerobacillus*
Bacteria; Firmicutes; Bacilli; Bacillales; Family XI Incertae Sedis; *Gemella*
Bacteria; Firmicutes; Bacilli; Bacillales; Paenibacillaceae; *Paenibacillus*
Bacteria; Firmicutes; Bacilli; Bacillales; Staphylococcaceae; Staphylococcus
Bacteria; Firmicutes; Bacilli; Lactobacillales
Bacteria; Firmicutes; Bacilli; Lactobacillales; Carnobacteriaceae
Bacteria; Firmicutes; Bacilli; Lactobacillales; Carnobacteriaceae; *Atopostipes*
Bacteria; Firmicutes; Bacilli; Lactobacillales; Carnobacteriaceae; *Granulicatella*
Bacteria; Firmicutes; Bacilli; Lactobacillales; Enterococcaceae; *Enterococcus*
Bacteria; Firmicutes; Bacilli; Lactobacillales; Lactobacillaceae; *Lactobacillus*

Bacteria; Firmicutes; Bacilli; Lactobacillales; Leuconostocaceae; *Leuconostoc*
Bacteria; Firmicutes; Bacilli; Lactobacillales; Streptococcaceae; *Streptococcus*
Bacteria; Firmicutes; Bacilli; VAN12
Bacteria; Firmicutes; Clostridia
Bacteria; Firmicutes; Clostridia; Candidatus *Desulforudis*
Bacteria; Firmicutes; Clostridia; Clostridiales
Bacteria; Firmicutes; Clostridia; Clostridiales; Clostridiaceae
Bacteria; Firmicutes; Clostridia; Clostridiales; Clostridiaceae; *Caloramator*
Bacteria; Firmicutes; Clostridia; Clostridiales; Clostridiaceae; *Clostridium*
Bacteria; Firmicutes; Clostridia; Clostridiales; Family XI Incertae Sedis; *Anaerococcus*
Bacteria; Firmicutes; Clostridia; Clostridiales; Family XI Incertae Sedis; *Peptoniphilus*
Bacteria; Firmicutes; Clostridia; Clostridiales; Family XVIII Incertae Sedis; *Symbiobacterium*
Bacteria; Firmicutes; Clostridia; Clostridiales; Family XVI Incertae Sedis; *Carboxydocella*
Bacteria; Firmicutes; Clostridia; Clostridiales; Lachnospiraceae
Bacteria; Firmicutes; Clostridia; Clostridiales; P. palm C-A 51
Bacteria; Firmicutes; Clostridia; Clostridiales; Peptococcaceae
Bacteria; Firmicutes; Clostridia; Clostridiales; Peptococcaceae; *Desulfitibacter*
Bacteria; Firmicutes; Clostridia; Clostridiales; Peptococcaceae; *Desulfotomaculum*
Bacteria; Firmicutes; Clostridia; Clostridiales; Peptococcaceae; *Desulfurispora*
Bacteria; Firmicutes; Clostridia; Clostridiales; Ruminococcaceae
Bacteria; Firmicutes; Clostridia; Clostridiales; Ruminococcaceae; *Incertae Sedis*
Bacteria; Firmicutes; Clostridia; Clostridiales; Syntrophomonadaceae; *Dethiobacter*
Bacteria; Firmicutes; Clostridia; Clostridiales; TSAC18
Bacteria; Firmicutes; Clostridia; D8A-2
Bacteria; Firmicutes; Clostridia; Thermoanaerobacterales; SRB2
Bacteria; Firmicutes; Clostridia; Thermoanaerobacterales; Thermoanaerobacteraceae; *Thermanaeromonas*
Bacteria; Firmicutes; Erysipelotrichi; Erysipelotrichales; Erysipelotrichaceae; *Erysipelothrix*
Bacteria; Fusobacteria; Fusobacteria; Fusobacteriales; Leptotrichiaceae; *Leptotrichia*
Bacteria; GOUTA4
Bacteria; Gemmatimonadetes; Gemmatimonadetes; BD2-11 terrestrial group
Bacteria; Gemmatimonadetes; Gemmatimonadetes; Gemmatimonadales; Gemmatimonadaceae
Bacteria; Gemmatimonadetes; Gemmatimonadetes; Gemmatimonadales; Gemmatimonadaceae
Bacteria; Kazan-3B-28
Bacteria; Lentisphaerae; Lentisphaeria; Victivallales; Victivallaceae
Bacteria; NPL-UPA2
Bacteria; Nitrospirae; Nitrospira; Nitrospirales; 0319-6A21
Bacteria; Nitrospirae; Nitrospira; Nitrospirales; 4-29
Bacteria; Nitrospirae; Nitrospira; Nitrospirales; Nitrospiraceae
Bacteria; Nitrospirae; Nitrospira; Nitrospirales; Nitrospiraceae; *Leptospirillum*
Bacteria; Nitrospirae; Nitrospira; Nitrospirales; Nitrospiraceae; *Nitrospira*
Bacteria; Nitrospirae; Nitrospira; Nitrospirales; Nitrospiraceae; *Thermodesulfovibrio*
Bacteria; Nitrospirae; Nitrospira; Nitrospirales; Nitrospiraceae
Bacteria; Nitrospirae; Nitrospira; Nitrospirales; OPB95
Bacteria; Planctomycetes
Bacteria; Planctomycetes; OM190
Bacteria; Planctomycetes; Phycisphaerae; CCM11a
Bacteria; Planctomycetes; Phycisphaerae; MSB-3A7 sediment group
Bacteria; Planctomycetes; Phycisphaerae; MSBL9
Bacteria; Planctomycetes; Phycisphaerae; Phycisphaerales; Phycisphaeraceae; AKYG587
Bacteria; Planctomycetes; Phycisphaerae; Phycisphaerales; Phycisphaeraceae; SM1A02
Bacteria; Planctomycetes; Phycisphaerae; S-70
Bacteria; Planctomycetes; Phycisphaerae; S15A-MN16
Bacteria; Planctomycetes; Phycisphaerae; WD2101 soil group
Bacteria; Planctomycetes; Pla4 lineage
Bacteria; Planctomycetes; Planctomycetacia; Brocadiales; Brocadiaceae; Candidatus *Brocadia*
Bacteria; Planctomycetes; Planctomycetacia; Planctomycetales; Planctomycetaceae
Bacteria; Planctomycetes; Planctomycetacia; Planctomycetales; Planctomycetaceae; *Blastopirellula*
Bacteria; Planctomycetes; Planctomycetacia; Planctomycetales; Planctomycetaceae; *Pirellula*
Bacteria; Planctomycetes; Planctomycetacia; Planctomycetales; Planctomycetaceae; *Planctomyces*
Bacteria; Planctomycetes; Planctomycetacia; Planctomycetales; Planctomycetaceae
Bacteria; Proteobacteria
Bacteria; Proteobacteria; Alphaproteobacteria
Bacteria; Proteobacteria; Alphaproteobacteria; 4-Org1-14
Bacteria; Proteobacteria; Alphaproteobacteria; Caulobacterales; Caulobacteraceae
Bacteria; Proteobacteria; Alphaproteobacteria; Caulobacterales; Caulobacteraceae; *Brevundimonas*
Bacteria; Proteobacteria; Alphaproteobacteria; Caulobacterales; Caulobacteraceae; *Caulobacter*
Bacteria; Proteobacteria; Alphaproteobacteria; Caulobacterales; Caulobacteraceae; *Phenylobacterium*
Bacteria; Proteobacteria; Alphaproteobacteria; Caulobacterales; Hyphomonadaceae; *Hirschia*
Bacteria; Proteobacteria; Alphaproteobacteria; Caulobacterales; Hyphomonadaceae; *Hyphomonas*
Bacteria; Proteobacteria; Alphaproteobacteria; Caulobacterales; Hyphomonadaceae; *Woodsholea*
Bacteria; Proteobacteria; Alphaproteobacteria; Caulobacterales; Hyphomonadaceae
Bacteria; Proteobacteria; Alphaproteobacteria; Rhizobiales
Bacteria; Proteobacteria; Alphaproteobacteria; Rhizobiales; 1174-901-12
Bacteria; Proteobacteria; Alphaproteobacteria; Rhizobiales; A0839
Bacteria; Proteobacteria; Alphaproteobacteria; Rhizobiales; Bradyrhizobiaceae
Bacteria; Proteobacteria; Alphaproteobacteria; Rhizobiales; Bradyrhizobiaceae; *Bradyrhizobium*
Bacteria; Proteobacteria; Alphaproteobacteria; Rhizobiales; Hyphomicrobiaceae
Bacteria; Proteobacteria; Alphaproteobacteria; Rhizobiales; Hyphomicrobiaceae; *Devosia*
Bacteria; Proteobacteria; Alphaproteobacteria; Rhizobiales; Hyphomicrobiaceae; *Hyphomicrobium*
Bacteria; Proteobacteria; Alphaproteobacteria; Rhizobiales; Hyphomicrobiaceae; *Pedomicrobium*
Bacteria; Proteobacteria; Alphaproteobacteria; Rhizobiales; MNG7
Bacteria; Proteobacteria; Alphaproteobacteria; Rhizobiales; Methylobacteriaceae; *Methylobacterium*
Bacteria; Proteobacteria; Alphaproteobacteria; Rhizobiales; Methylobacteriaceae
Bacteria; Proteobacteria; Alphaproteobacteria; Rhizobiales; Methylocystaceae; *Methylocystis*
Bacteria; Proteobacteria; Alphaproteobacteria; Rhizobiales; Phyllobacteriaceae
Bacteria; Proteobacteria; Alphaproteobacteria; Rhizobiales; Rhodobiaceae; *Parvibaculum*
Bacteria; Proteobacteria; Alphaproteobacteria; Rhizobiales; Xanthobacteraceae
Bacteria; Proteobacteria; Alphaproteobacteria; Rhodobacterales; Rhodobacteraceae
Bacteria; Proteobacteria; Alphaproteobacteria; Rhodobacterales; Rhodobacteraceae; *Paracoccus*
Bacteria; Proteobacteria; Alphaproteobacteria; Rhodobacterales; Rhodobacteraceae; *Pseudorhodobacter*
Bacteria; Proteobacteria; Alphaproteobacteria; Rhodobacterales; Rhodobacteraceae; *Rubellimicrobium*
Bacteria; Proteobacteria; Alphaproteobacteria; Rhodospirillales; Acetobacteraceae
Bacteria; Proteobacteria; Alphaproteobacteria; Rhodospirillales; Acetobacteraceae; *Roseococcus*
Bacteria; Proteobacteria; Alphaproteobacteria; Rhodospirillales; Acetobacteraceae; *Roseomonas*
Bacteria; Proteobacteria; Alphaproteobacteria; Rhodospirillales; CCU22
Bacteria; Proteobacteria; Alphaproteobacteria; Rhodospirillales; DA111
Bacteria; Proteobacteria; Alphaproteobacteria; Rhodospirillales; Family Incertae Sedis; *Oleomonas*
Bacteria; Proteobacteria; Alphaproteobacteria; Rhodospirillales; I-10
Bacteria; Proteobacteria; Alphaproteobacteria; Rhodospirillales; KCM-B-60
Bacteria; Proteobacteria; Alphaproteobacteria; Rhodospirillales; Rhodospirillaceae
Bacteria; Proteobacteria; Alphaproteobacteria; Rhodospirillales; Rhodospirillaceae; *Dongia*
Bacteria; Proteobacteria; Alphaproteobacteria; Rhodospirillales; Rhodospirillaceae; *Magnetospirillum*
Bacteria; Proteobacteria; Alphaproteobacteria; Rhodospirillales; Rhodospirillaceae; *Skermanella*
Bacteria; Proteobacteria; Alphaproteobacteria; Rhodospirillales; wr0007
Bacteria; Proteobacteria; Alphaproteobacteria; Rickettsiales
Bacteria; Proteobacteria; Alphaproteobacteria; Rickettsiales; Candidatus *Captivus*
Bacteria; Proteobacteria; Alphaproteobacteria; Rickettsiales; Holosporaceae; *Holospora*
Bacteria; Proteobacteria; Alphaproteobacteria; Rickettsiales; Rickettsiaceae; *Rickettsia*
Bacteria; Proteobacteria; Alphaproteobacteria; Rickettsiales; Rickettsiaceae
Bacteria; Proteobacteria; Alphaproteobacteria; Rickettsiales; SM2D12
Bacteria; Proteobacteria; Alphaproteobacteria; Rickettsiales; mitochondria
Bacteria; Proteobacteria; Alphaproteobacteria; SB1-18
Bacteria; Proteobacteria; Alphaproteobacteria; Sphingomonadales
Bacteria; Proteobacteria; Alphaproteobacteria; Sphingomonadales; Erythrobacteraceae
Bacteria; Proteobacteria; Alphaproteobacteria; Sphingomonadales; Erythrobacteraceae; *Altererythrobacter*
Bacteria; Proteobacteria; Alphaproteobacteria; Sphingomonadales; GOBB3-C201
Bacteria; Proteobacteria; Alphaproteobacteria; Sphingomonadales; Sphingomonadaceae
Bacteria; Proteobacteria; Alphaproteobacteria; Sphingomonadales; Sphingomonadaceae; *Sphingobium*
Bacteria; Proteobacteria; Alphaproteobacteria; Sphingomonadales; Sphingomonadaceae; *Sphingomonas*
Bacteria; Proteobacteria; Alphaproteobacteria; Sphingomonadales; Sphingomonadaceae; *Sphingopyxis*
Bacteria; Proteobacteria; Alphaproteobacteria; Sphingomonadales; Sphingomonadaceae; *Sphingosinicella*
Bacteria; Proteobacteria; Betaproteobacteria
Bacteria; Proteobacteria; Betaproteobacteria; Burkholderiales
Bacteria; Proteobacteria; Betaproteobacteria; Burkholderiales; Alcaligenaceae
Bacteria; Proteobacteria; Betaproteobacteria; Burkholderiales; Burkholderiaceae; *Cupriavidus*
Bacteria; Proteobacteria; Betaproteobacteria; Burkholderiales; Burkholderiaceae; *Ralstonia*
Bacteria; Proteobacteria; Betaproteobacteria; Burkholderiales; Comamonadaceae
Bacteria; Proteobacteria; Betaproteobacteria; Burkholderiales; Comamonadaceae; *Alicycliphilus*
Bacteria; Proteobacteria; Betaproteobacteria; Burkholderiales; Comamonadaceae; *Aquabacterium*
Bacteria; Proteobacteria; Betaproteobacteria; Burkholderiales; Comamonadaceae; *Caldimonas*
Bacteria; Proteobacteria; Betaproteobacteria; Burkholderiales; Comamonadaceae; *Diaphorobacter*
Bacteria; Proteobacteria; Betaproteobacteria; Burkholderiales; Comamonadaceae; *Polaromonas*
Bacteria; Proteobacteria; Betaproteobacteria; Burkholderiales; Comamonadaceae; *Ramlibacter*
Bacteria; Proteobacteria; Betaproteobacteria; Burkholderiales; Comamonadaceae; *Rhizobacter*
Bacteria; Proteobacteria; Betaproteobacteria; Burkholderiales; Comamonadaceae; *Schlegelella*
Bacteria; Proteobacteria; Betaproteobacteria; Burkholderiales; Comamonadaceae; *Tepidicella*
Bacteria; Proteobacteria; Betaproteobacteria; Burkholderiales; Comamonadaceae; *Variovorax*
Bacteria; Proteobacteria; Betaproteobacteria; Burkholderiales; Oxalobacteraceae
Bacteria; Proteobacteria; Betaproteobacteria; Burkholderiales; Oxalobacteraceae; *Undibacterium*
Bacteria; Proteobacteria; Betaproteobacteria; Hydrogenophilales; Hydrogenophilaceae
Bacteria; Proteobacteria; Betaproteobacteria; Hydrogenophilales; Hydrogenophilaceae; *Sulfuricella*
Bacteria; Proteobacteria; Betaproteobacteria; Hydrogenophilales; Hydrogenophilaceae; *Thiobacillus*
Bacteria; Proteobacteria; Betaproteobacteria; Hydrogenophilales; Hydrogenophilaceae
Bacteria; Proteobacteria; Betaproteobacteria; Methylophilales; Methylophilaceae
Bacteria; Proteobacteria; Betaproteobacteria; Methylophilales; Methylophilaceae
Bacteria; Proteobacteria; Betaproteobacteria; Neisseriales; Neisseriaceae
Bacteria; Proteobacteria; Betaproteobacteria; Neisseriales; Neisseriaceae
Bacteria; Proteobacteria; Betaproteobacteria; Nitrosomonadales; Gallionellaceae
Bacteria; Proteobacteria; Betaproteobacteria; Nitrosomonadales; Gallionellaceae; Candidatus *Nitrotoga*
Bacteria; Proteobacteria; Betaproteobacteria; Nitrosomonadales; Gallionellaceae; *Sideroxydans*
Bacteria; Proteobacteria; Betaproteobacteria; Nitrosomonadales; Nitrosomonadaceae; *Nitrosospira*
Bacteria; Proteobacteria; Betaproteobacteria; Nitrosomonadales; Nitrosomonadaceae
Bacteria; Proteobacteria; Betaproteobacteria; Rhodocyclales; Rhodocyclaceae
Bacteria; Proteobacteria; Betaproteobacteria; Rhodocyclales; Rhodocyclaceae; *Ferribacterium*
Bacteria; Proteobacteria; Betaproteobacteria; Rhodocyclales; Rhodocyclaceae; *Methyloversatilis*
Bacteria; Proteobacteria; Betaproteobacteria; Rhodocyclales; Rhodocyclaceae; *Sterolibacterium*
Bacteria; Proteobacteria; Betaproteobacteria; Rhodocyclales; Rhodocyclaceae; *Zoogloea*
Bacteria; Proteobacteria; Betaproteobacteria; Rhodocyclales; Rhodocyclaceae
Bacteria; Proteobacteria; Betaproteobacteria; TRA3-20
Bacteria; Proteobacteria; Deltaproteobacteria
Bacteria; Proteobacteria; Deltaproteobacteria; 43F-1404R
Bacteria; Proteobacteria; Deltaproteobacteria; Bdellovibrionales; Bacteriovoracaceae; *Bacteriovorax*
Bacteria; Proteobacteria; Deltaproteobacteria; Bdellovibrionales; Bacteriovoracaceae; *Peredibacter*
Bacteria; Proteobacteria; Deltaproteobacteria; Bdellovibrionales; Bdellovibrionaceae; *Bdellovibrio*
Bacteria; Proteobacteria; Deltaproteobacteria; Bdellovibrionales; Bdellovibrionaceae; OM27 clade
Bacteria; Proteobacteria; Deltaproteobacteria; DTB120
Bacteria; Proteobacteria; Deltaproteobacteria; Desulfobacterales; Desulfobacteraceae
Bacteria; Proteobacteria; Deltaproteobacteria; Desulfobacterales; Nitrospinaceae
Bacteria; Proteobacteria; Deltaproteobacteria; Desulfovibrionales; Desulfomicrobiaceae; *Desulfomicrobium*
Bacteria; Proteobacteria; Deltaproteobacteria; Desulfovibrionales; Desulfovibrionaceae; *Desulfovibrio*
Bacteria; Proteobacteria; Deltaproteobacteria; Desulfurellales; Desulfurellaceae
Bacteria; Proteobacteria; Deltaproteobacteria; Desulfuromonadales
Bacteria; Proteobacteria; Deltaproteobacteria; Desulfuromonadales; GR-WP33-58
Bacteria; Proteobacteria; Deltaproteobacteria; GR-WP33-30
Bacteria; Proteobacteria; Deltaproteobacteria; Myxococcales
Bacteria; Proteobacteria; Deltaproteobacteria; Myxococcales; 0319-6G20
Bacteria; Proteobacteria; Deltaproteobacteria; Myxococcales; Cystobacterineae
Bacteria; Proteobacteria; Deltaproteobacteria; Myxococcales; Haliangiaceae; *Haliangium*
Bacteria; Proteobacteria; Deltaproteobacteria; Myxococcales; Nannocystineae
Bacteria; Proteobacteria; Deltaproteobacteria; Myxococcales; Sandaracinaceae; *Sandaracinus*
Bacteria; Proteobacteria; Deltaproteobacteria; Myxococcales; Sorangiineae; Sandaracinaceae
Bacteria; Proteobacteria; Deltaproteobacteria; Myxococcales; mle1-27
Bacteria; Proteobacteria; Deltaproteobacteria; Sh765B-TzT-29
Bacteria; Proteobacteria; Deltaproteobacteria; Sva0485
Bacteria; Proteobacteria; Deltaproteobacteria; Syntrophobacterales; Syntrophaceae
Bacteria; Proteobacteria; Deltaproteobacteria; Syntrophobacterales; Syntrophaceae; *Syntrophus*
Bacteria; Proteobacteria; Epsilonproteobacteria; Campylobacterales; Campylobacteraceae; *Campylobacter*
Bacteria; Proteobacteria; Epsilonproteobacteria; Campylobacterales; Helicobacteraceae; *Sulfuricurvum*
Bacteria; Proteobacteria; Epsilonproteobacteria; Campylobacterales; Helicobacteraceae; *Sulfurimonas*
Bacteria; Proteobacteria; Gammaproteobacteria
Bacteria; Proteobacteria; Gammaproteobacteria; 34P16
Bacteria; Proteobacteria; Gammaproteobacteria; Acidithiobacillales; KCM-B-112
Bacteria; Proteobacteria; Gammaproteobacteria; Aeromonadales; Aeromonadaceae
Bacteria; Proteobacteria; Gammaproteobacteria; Alteromonadales; Alteromonadaceae; C1-B045
Bacteria; Proteobacteria; Gammaproteobacteria; Alteromonadales; Alteromonadaceae; *Marinobacter*
Bacteria; Proteobacteria; Gammaproteobacteria; Alteromonadales; Colwelliaceae; *Colwellia*
Bacteria; Proteobacteria; Gammaproteobacteria; Alteromonadales; Shewanellaceae; *Shewanella*
Bacteria; Proteobacteria; Gammaproteobacteria; Chromatiales
Bacteria; Proteobacteria; Gammaproteobacteria; Chromatiales; Chromatiaceae; *Rheinheimera*
Bacteria; Proteobacteria; Gammaproteobacteria; Chromatiales; Ectothiorhodospiraceae; *Acidiferrobacter*
Bacteria; Proteobacteria; Gammaproteobacteria; Enterobacteriales; Enterobacteriaceae
Bacteria; Proteobacteria; Gammaproteobacteria; Enterobacteriales; Enterobacteriaceae; *Klebsiella*
Bacteria; Proteobacteria; Gammaproteobacteria; Legionellales; Coxiellaceae; *Aquicella*
Bacteria; Proteobacteria; Gammaproteobacteria; Legionellales; Coxiellaceae; *Coxiella*
Bacteria; Proteobacteria; Gammaproteobacteria; Legionellales; Coxiellaceae
Bacteria; Proteobacteria; Gammaproteobacteria; Legionellales; Legionellaceae; *Legionella*
Bacteria; Proteobacteria; Gammaproteobacteria; Methylococcales
Bacteria; Proteobacteria; Gammaproteobacteria; Methylococcales; IheB2-23
Bacteria; Proteobacteria; Gammaproteobacteria; Methylococcales; Methylococcaceae; *Methylobacter*
Bacteria; Proteobacteria; Gammaproteobacteria; Methylococcales; Methylococcaceae; *Methylocaldum*
Bacteria; Proteobacteria; Gammaproteobacteria; NKB5
Bacteria; Proteobacteria; Gammaproteobacteria; Oceanospirillales
Bacteria; Proteobacteria; Gammaproteobacteria; Oceanospirillales; Halomonadaceae; *Halomonas*
Bacteria; Proteobacteria; Gammaproteobacteria; Oceanospirillales; Oceanospirillaceae; *Pseudospirillum*
Bacteria; Proteobacteria; Gammaproteobacteria; Pasteurellales; Pasteurellaceae; *Haemophilus*
Bacteria; Proteobacteria; Gammaproteobacteria; Pseudomonadales; Moraxellaceae; *Acinetobacter*
Bacteria; Proteobacteria; Gammaproteobacteria; Pseudomonadales; Moraxellaceae; *Alkanindiges*
Bacteria; Proteobacteria; Gammaproteobacteria; Pseudomonadales; Moraxellaceae; *Enhydrobacter*
Bacteria; Proteobacteria; Gammaproteobacteria; Pseudomonadales; Moraxellaceae; *Perlucidibaca*
Bacteria; Proteobacteria; Gammaproteobacteria; Pseudomonadales; Moraxellaceae
Bacteria; Proteobacteria; Gammaproteobacteria; Pseudomonadales; Pseudomonadaceae; *Pseudomonas*
Bacteria; Proteobacteria; Gammaproteobacteria; Thiotrichales; CHAB-XI-27
Bacteria; Proteobacteria; Gammaproteobacteria; Thiotrichales; Thiotrichaceae; *Beggiatoa*
Bacteria; Proteobacteria; Gammaproteobacteria; Vibrionales; Vibrionaceae
Bacteria; Proteobacteria; Gammaproteobacteria; Xanthomonadales; Sinobacteraceae; *Nevskia*
Bacteria; Proteobacteria; Gammaproteobacteria; Xanthomonadales; Sinobacteraceae
Bacteria; Proteobacteria; Gammaproteobacteria; Xanthomonadales; Xanthomonadaceae
Bacteria; Proteobacteria; Gammaproteobacteria; Xanthomonadales; Xanthomonadaceae; *Aquimonas*
Bacteria; Proteobacteria; Gammaproteobacteria; Xanthomonadales; Xanthomonadaceae; *Lysobacter*
Bacteria; Proteobacteria; Gammaproteobacteria; Xanthomonadales; Xanthomonadaceae; *Pseudoxanthomonas*
Bacteria; Proteobacteria; Gammaproteobacteria; Xanthomonadales; Xanthomonadaceae
Bacteria; Proteobacteria; SC3-20
Bacteria; SM2F11
Bacteria; Spirochaetes; Spirochaetes; Spirochaetales; Leptospiraceae; *Turneriella*
Bacteria; Spirochaetes; Spirochaetes; Spirochaetales; PL-11B10
Bacteria; Spirochaetes; Spirochaetes; Spirochaetales; Spirochaetaceae; *Spirochaeta*
Bacteria; TA06
Bacteria; TM6
Bacteria; Thermotogae; Thermotogae; Thermotogales; Thermotogaceae; GAL15
Bacteria; Verrucomicrobia
Bacteria; Verrucomicrobia; OPB35 soil group
Bacteria; Verrucomicrobia; OPB35 soil group
Bacteria; Verrucomicrobia; Opitutae
Bacteria; Verrucomicrobia; Opitutae; Opitutales; Opitutaceae; *Opitutus*
Bacteria; Verrucomicrobia; Opitutae
Bacteria; Verrucomicrobia; S-BQ2-57 soil group
Bacteria; Verrucomicrobia; Spartobacteria; Chthoniobacterales
Bacteria; Verrucomicrobia; Spartobacteria; Chthoniobacterales; Chthoniobacteraceae; *Chthoniobacter*
Bacteria; Verrucomicrobia; Verrucomicrobiae; Verrucomicrobiales; Verrucomicrobiaceae; *Prosthecobacter*
None
Unclassified

Supplementary Figure 5. Relative proportions of the taxa retrieved in duplicates by 454-pyrosequencing of the bacterial 16S-rRNA gene sequences in groundwater from control well HN-01 and monitoring well HN-04. It can be observed that for two successive bars that correspond to replicates, results are in good agreement.

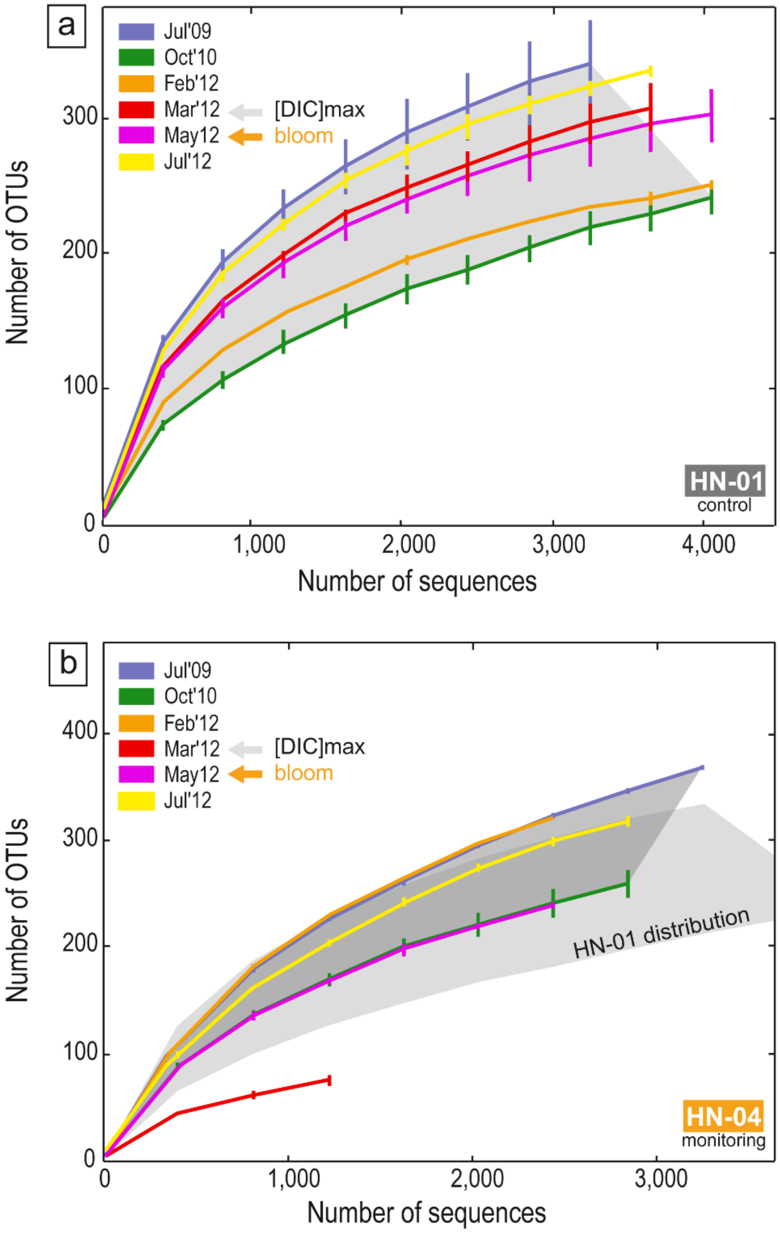

Supplementary Figure 6. Relative bacterial richness from wells HN-01 (a) and HN-04 (b) shown through rarefaction analysis (based on 454-pyrosequencing data). Operational Taxonomic Units (OTUs) were defined at a sequence similarity ≥ 97%. Rarefaction curves revealed a large bacterial diversity for both wells at all sampling periods except for the bacterial community sampled in March 2012 (Mar'12) in HN-04 groundwater, when the fast flowing fraction of pure CO_2 acidified the aquifer close to HN-04 with a marked effect on bacterial richness (see also Figs. 1c-d).

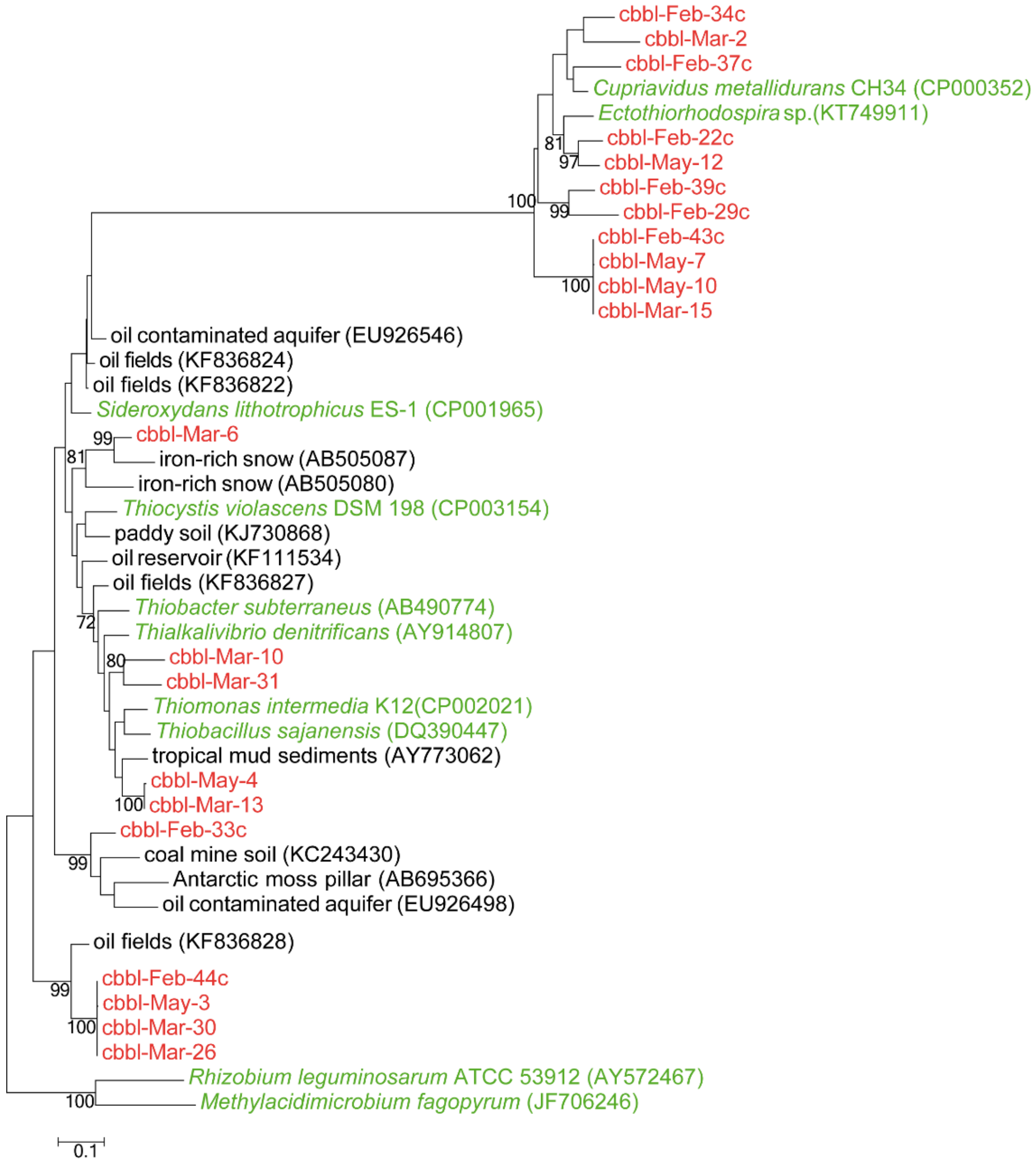

Supplementary Figure 7. Maximum likelihood phylogenetic tree of the *cbbL* gene sequences amplified by PCR from groundwater of HN-04 monitoring well sampled in February (cbbl-Feb), March (cbbl-Mar) and May (cbbl-May) 2012. Clones from this study and the closest cultivated strains are indicated in red and green, respectively. The tree was reconstructed by using the Maximum Likelihood method based on the Jukes-Cantor[13] model, using MEGA6 software[14]. Only bootstrap values for nodes with > 70% support are displayed as percentages. *Cupriavidus* and *Thiomonas* species: respectively Betaproteobacteria of the Burkholderiaceae and Comamonadaceae families in the Burkholderiales order; *Sideroxydans* species: Betaproteobacteria of the Gallionellaceae family in the Gallionellales order; *Thiobacter* species: Betaproteobacteria of the Burkholderiales order; *Thiobacillus* species: Betaproteobacteria of the Hydrogenophilales order; *Ectothiorhodospira*, *Thiocystis* and *Thialkalivibrio* species: Gammaproteobacteria of the Chromatiales order; *Rhizobium* species: Alphaproteobacteria of the Rhizobiales order; *Methylacidimicrobium* species: Verrucomicrobia.

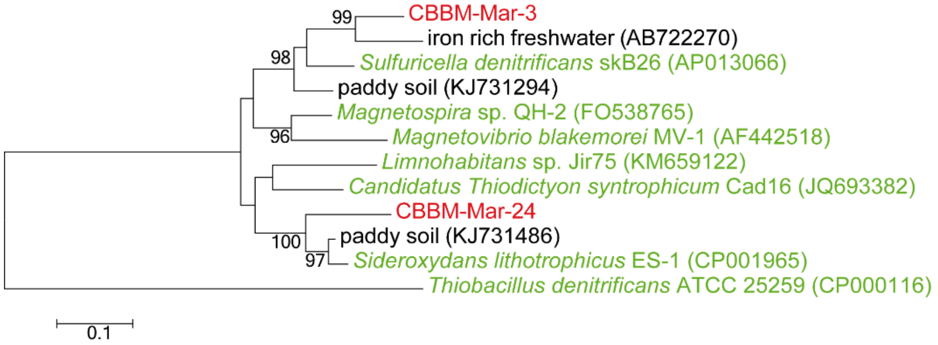

Supplementary Figure 8. Maximum likelihood phylogenetic tree of the *cbbM* gene sequences amplified by PCR from groundwater of HN-04 monitoring well sampled in March (CBBM-Mar) 2012. Clones from this study and the closest cultivated strains are indicated in red and green, respectively. The tree was reconstructed by using the Maximum Likelihood method based on the Jukes-Cantor[13] model, using MEGA6 software[14]. Only bootstrap values for nodes with > 70% support are displayed as percentages. *Sideroxydans* species: Betaproteobacteria of the Gallionellaceae family in the Gallionellales order; *Limnohabitans* species: Betaproteobacteria of the Burkholderiales order (Comamonadaceae family); *Thiobacillus* and *Sulfuricella* species: Betaproteobacteria of the Hydrogenophilales order; *Thiodictyon* species: Gammaproteobacteria of the Chromatiales order; *Magnetospira* and *Magnetovibrio* species: Alphaproteobacteria of the Rhodospirillales order.

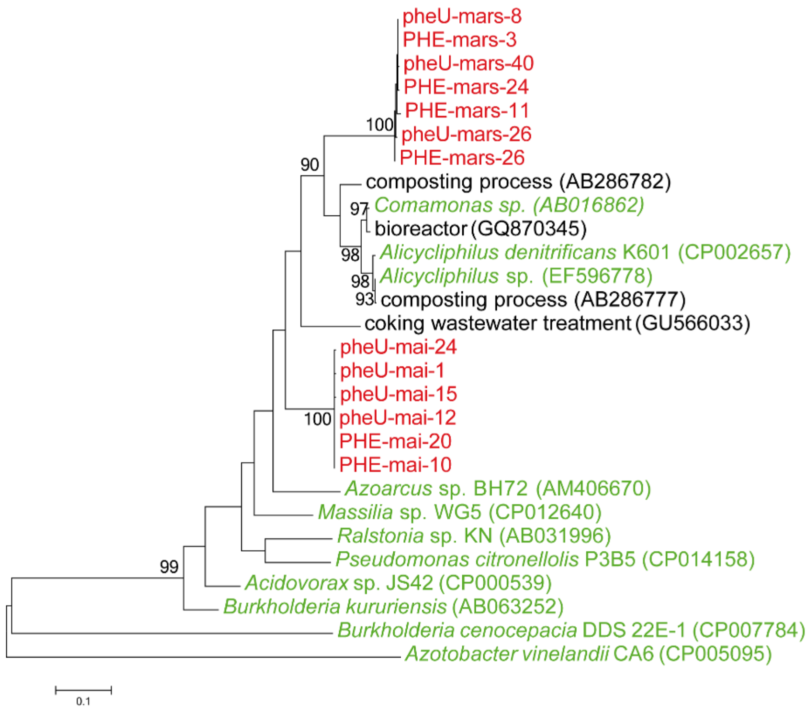

Supplementary Figure 9. Maximum likelihood phylogenetic tree of the gene sequences coding for the largest subunit of multicomponent phenol hydroxylases involved in phenolic compounds' degradation. Those were amplified by PCR from groundwater of HN-04 monitoring well sampled in March (pheU-mars and PHE-mars) and May (pheU-mai and PHE-mai) 2012. Clones from this study and the closest cultivated strains are indicated in red and green, respectively. The tree was reconstructed by using the Maximum Likelihood method based on the Jukes-Cantor[13] model, using MEGA6 software[14]. Only bootstrap values for nodes with > 70% support are displayed as percentages. *Comamonas*, *Alicycliphilus* and *Acidovorax* species: Betaproteobacteria of the Burkholderiales order (Comamonadaceae family); *Burkholderia* and *Ralstonia* species: Betaproteobacteria of the Burkholderiales order (Burkholderiaceae family); *Azoarcus* species: Betaproteobacteria of the Burkholderiales order (Rhodocyclaceae family); *Massilia* species: Betaproteobacteria of the Burkholderiales order (Oxalobacteraceae family); *Pseudomonas* and *Azotobacter* species: Gammaproteobacteria of the Pseudomonadales order.

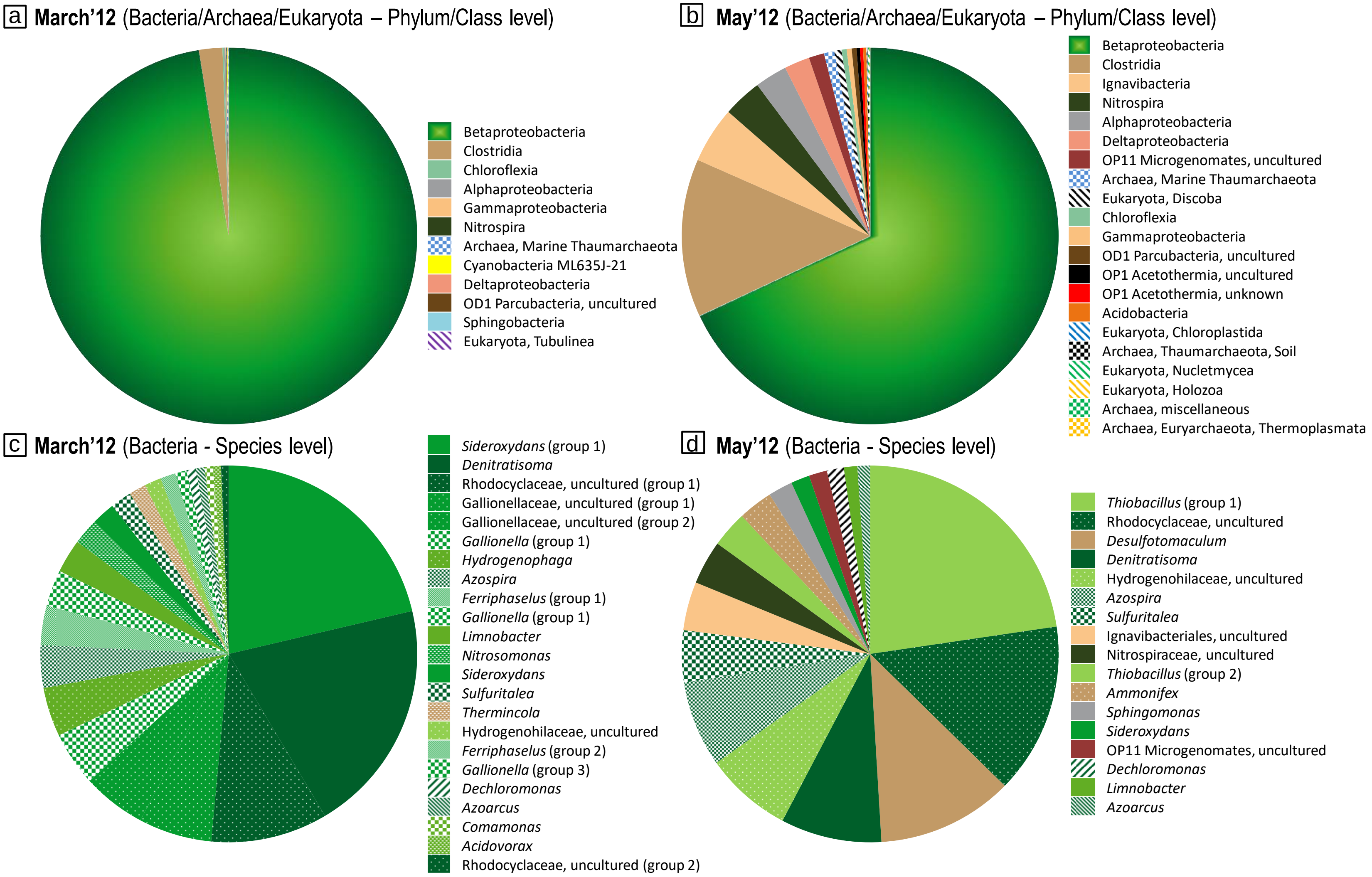

Supplementary Figure 10. Diversity analyses obtained from metagenomic data (rRNA gene) collected on HN-04 groundwater sampled in March and May 2012. They notably show the clear dominance of bacteria in HN-04 groundwater. Note that they match the 454-pyrosequencing results (Fig. 3).

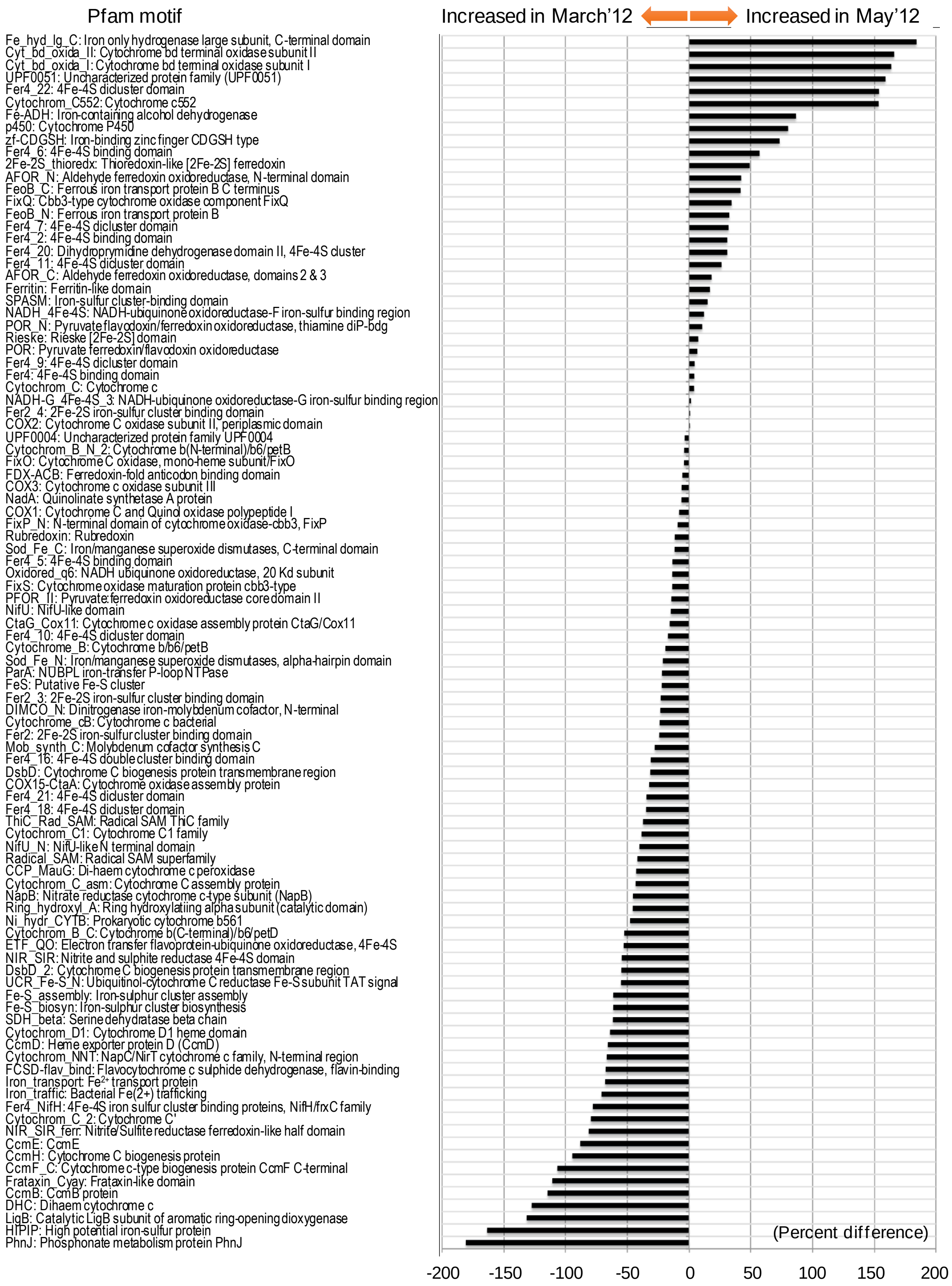

Supplementary Figure 11. Fe-related Pfam protein sequence motifs (in percent difference) obtained from metagenomic data collected on HN-04 groundwater sampled in March and May 2012.

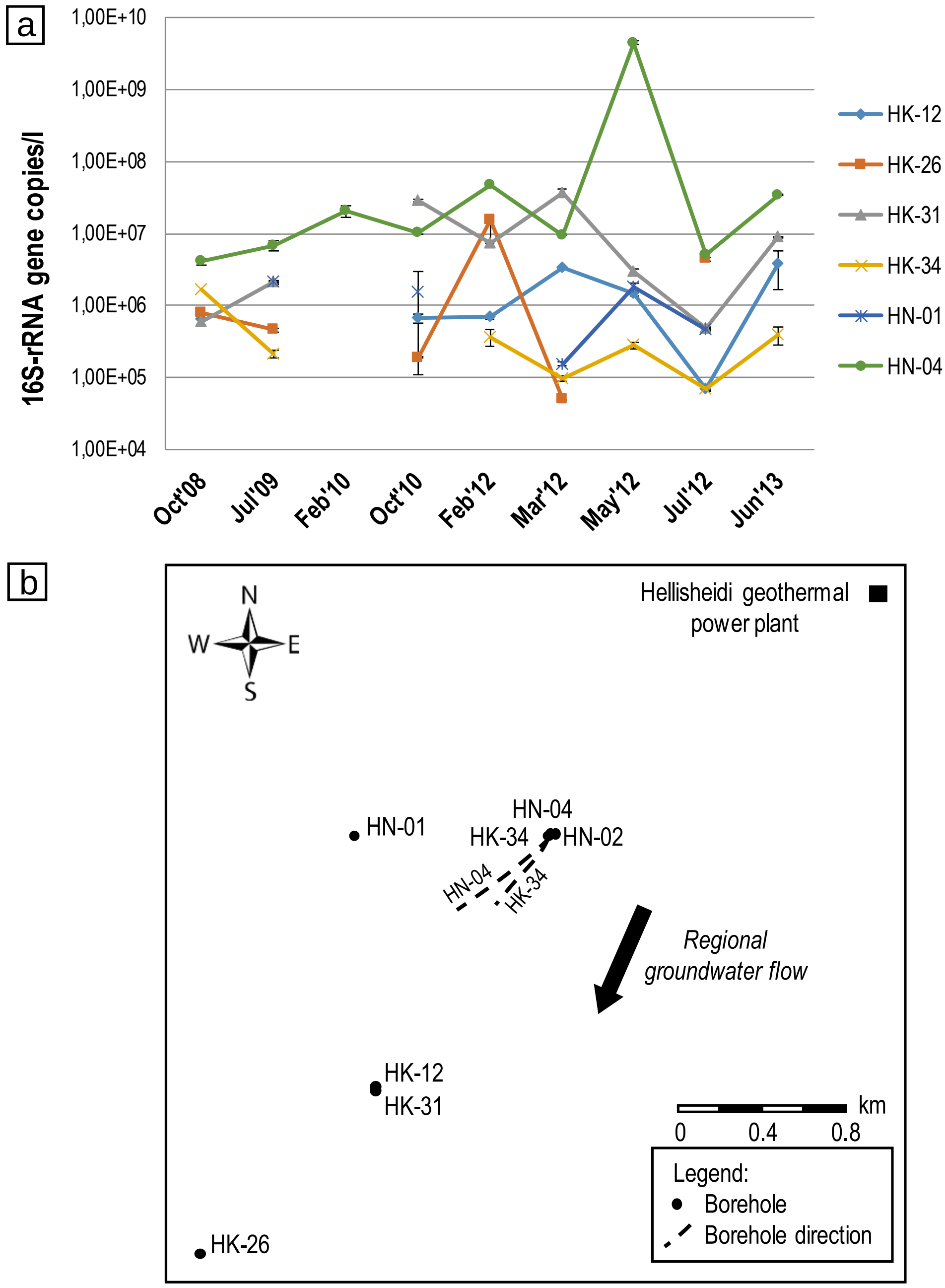

Supplementary Figure 12. (a) 16S-rRNA gene abundance obtained using qPCR for Bacteria in groundwater collected through the network of control and monitoring wells. Well locations are shown in **(b)** (map reproduced from Alfredsson et al., 2013[1]). Regional groundwater flows from NNE to SSW.

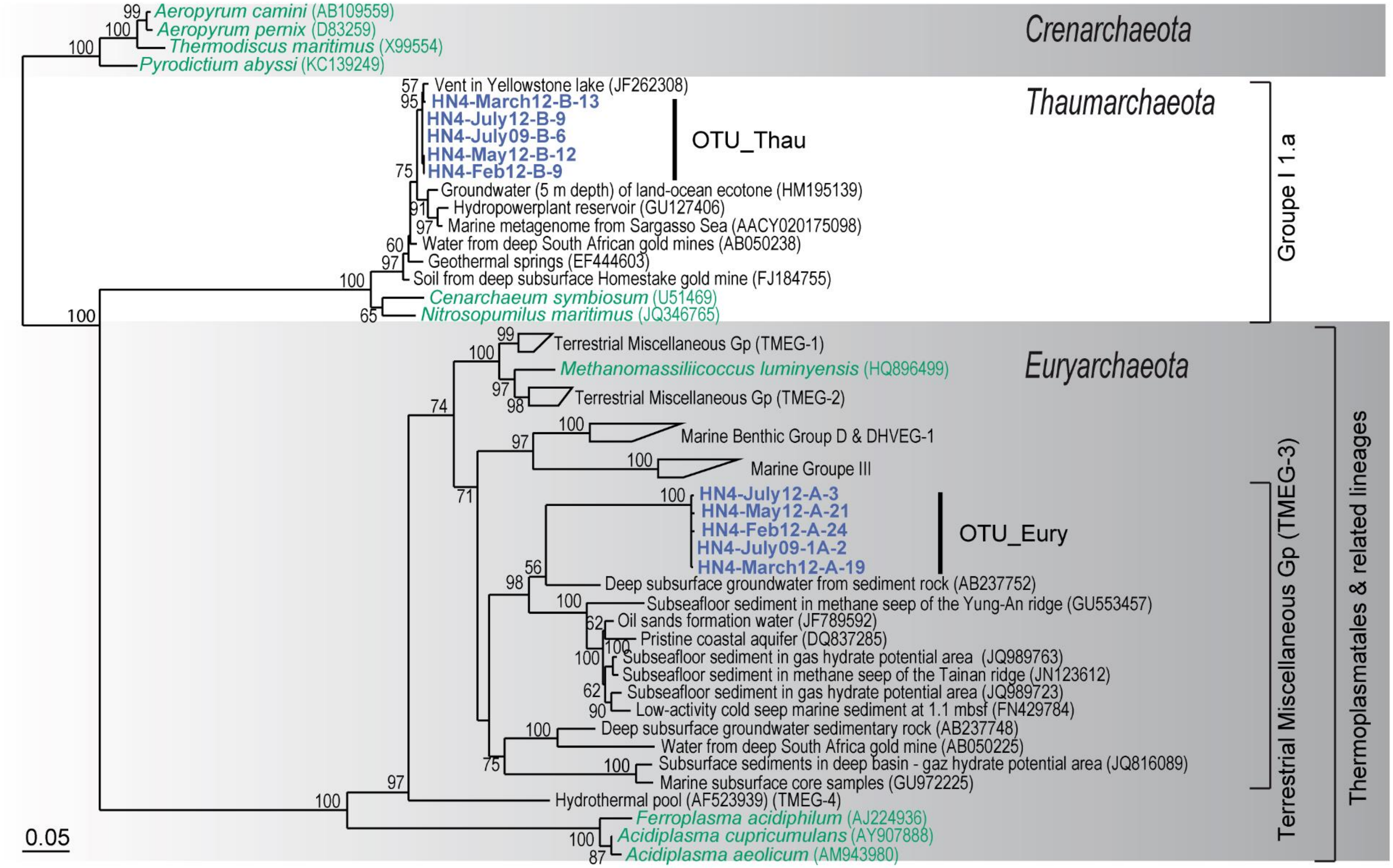

Supplementary Figure 13. Maximum likelihood phylogenetic tree showing the only two archaeal OTUs present all along the microbiological survey. The tree was reconstructed using the ARB software[15]. Clones from this study are indicated in blue and bold (full clone names as well # – sampling period # – primer # – archaeal clone #, e.g. HN4-July12-B-9). A and B stand for the Euryarchaeota-specific primer ANMEF and the Archaea-specific primer Ar109, respectively. The closest cultivated strains are indicated in green. Bootstrap values for nodes with > 50% support, based on 1,000 replicates, are displayed as percentages. The scale bar represents 0.05 substitutions per nucleotide position. The two

single OTUs belong to the Thaumarchaeota (OTU_Thaum)[16] and the Euryarchaeota (OTU_Eury) phyla. They form clades with other environmental sequences, but distantly relate to sequences of cultivated species (respectively 91 and 92% identities at the level of their partial 16S-rRNA gene sequences with *Nitrosopumilus maritimus* and *Crenarchaeum symbiosum* for the OTU_Thaum and 85% with *Methanomassiliicoccus luminyensis* for the OTU_Eury). The OTU_Thaum closely relates to a sequence retrieved from Yellowstone vent[17] and clusters with a group of sequences mainly retrieved from subsurface environments, including deep sea waters, groundwater, water or soil from subsurface gold mine, geothermal spring, and a hydropower reservoir. The OTU_Eury belongs to a clade that consists exclusively of environmental sequences, branching as a Thermoplasmatales-related group. The closest neighbor sequences were retrieved from deep subsurface groundwater collected in sedimentary rocks and other related sequences are from oil sand formation water, subseafloor sediments with gas hydrate potential, and petroleum reservoir. They are part of a Terrestrial Miscellaneous Euryarchaeotal Group[18] (i.e. TMEG-3).

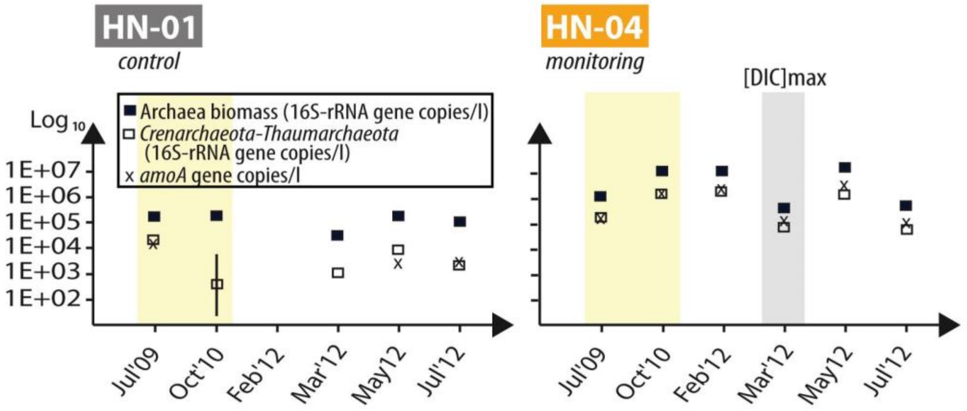

Supplementary Figure 14. Temporal evolution in groundwater of monitoring well HN-04 and control well HN-01 of gene abundance obtained using quantitative polymerase chain reaction (qPCR) for Archaea, Crenarchaeota-Thaumarchaeota and *amoA* gene. Note that, as no Crenarchaeota were detected by Sanger sequencing, the Crenarchaeota-Thaumarchaeota 16S-rRNA gene only represents Thaumarchaeota. The yellow boxes frame the pre-injection measurements whereas the gray box indicates the main arrival of the plume of pure CO_2 at the level of well HN-04 in March 2012.

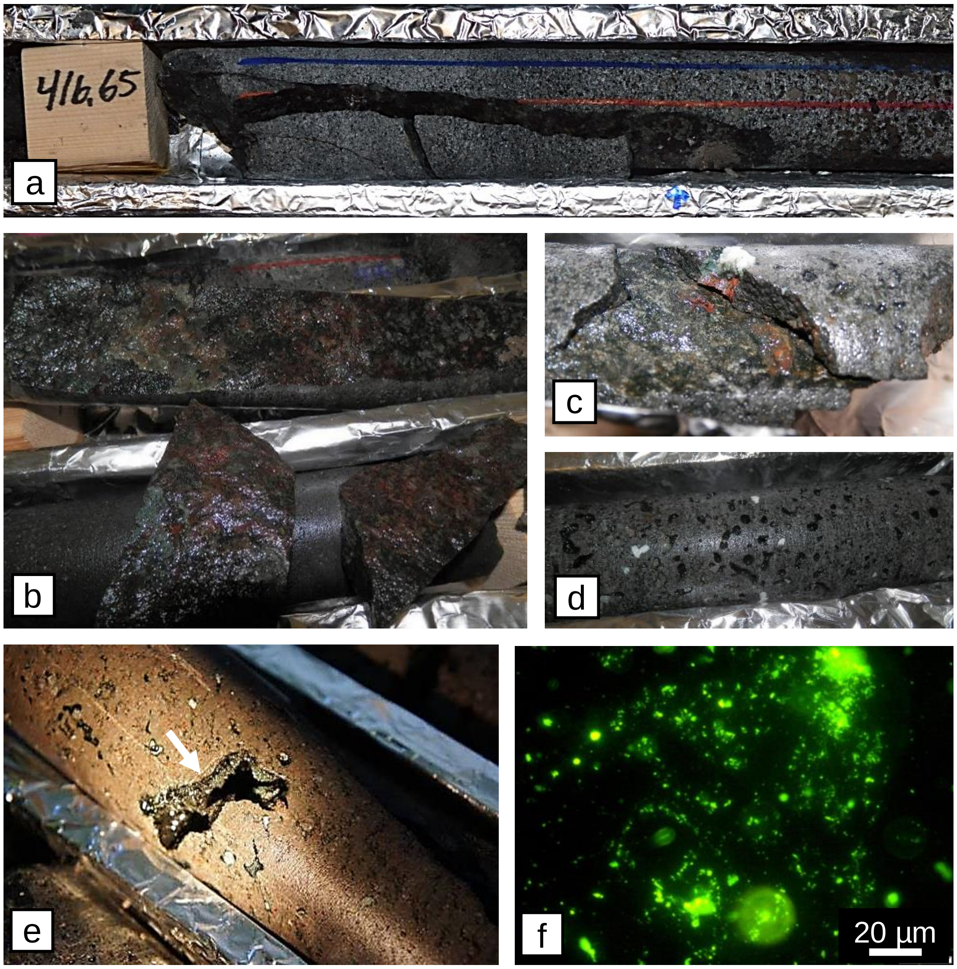

Supplementary Figure 15. Microphotographs of the core KB01 drilled in the storage formation in October 2014 (core diameter: 47.6 mm). The core well was established ca. halfway between injection well HN-02 and first monitoring well HN-04 (Supplementary Fig. 1). During drilling, feedzones were evidenced at ~420 m depth likely providing the fast flow pathway between HN-02 and HN-04 followed by the unreacted CO_2 detected in March 2012 in HN-04 (Supplementary Fig. 2a). (**a**) view of the basaltic rock at 416.65 m depth showing a large fracture crossing longitudinally the core. Both sides of the fracture plans are shown in (**b**) and evidenced secondary greenish to reddish coatings made of clays and iron oxides embedded in a slimy biofilm-like substance. (**c**) similar occurrences at ~440 m depths. (**d**) enlarged and interconnected vesicles in a core section collected at ~460 m depth and typifying the aquifer matrix porosity after injection. These vesicles were frequently filled with a greenish and slimy assemblage ± whitish mineral phases being either zeolites or carbonates. Vesicles were sometimes unusually large (> 1 cm in diameter) as the one shown by a white arrow on the core section retrieved at 479.09 m depth (**e**). After unspecific staining with Syto®9 dye, epifluorescence microscopy revealed the presence of a high microbial cell density in this slimy material (**f**). The extensive coating of iron (hydr)oxides could result from the iron oxidation activity of the autotrophic Gallionellaceae and *Thiobacillus* bacteria that bloomed when CO_2 and Fe concentrations increased in the aquifer following the gas injection (Supplementary Fig. 10). The low-density Fe-oxide/clay/mat material looks similar to the one commonly found surrounding oceanic basalts and formed from low-temperature seeps involving Fe-oxidizers[19-22].

Supplementary Table 1. Characteristics of the slug-type tracer tests and gas injections carried out at the Carbfix1 storage site.

	Slug-type tracer tests		Gas injection phases[7]	
	**pump-assisted test[8]**	**natural gradient test[2]**	**pure CO_2**	**geothermal gas mixture (75% CO_2-24.2% H_2S-0.8% H_2)**
**Period**	November 2007 to May 2008	June 2008 to June 2014	January, 24 to March, 9 2012 - 40 active days over 40	June, 15 to August, 1st 2012 - 29 active days over 48
**Mass of injected gas**	/	/	175 t	73 t
**Reactive tracers**	/	/	^{14}C (40.0 $Bq \cdot l^{-1}$)	^{14}C (6 $Bq \cdot l^{-1}$)
**Non-reactive tracers**	**Na-fluorescein (Na-Flu)** 0.5 kg	**Na-fluorescein (Na-Flu)** 50 kg **sulfurhexafluoride (SF_6)**	**sulfurhexafluoride (SF_6)** $2.33 \cdot 10^{-8}$ $ccSTP \cdot cc^{-1}$	**trifluoromethyl sulfur pentafluoride (SF_5CF_3)** $2.24 \cdot 10^{-8}$ $ccSTP \cdot cc^{-1}$

Supplementary Table 2. Major and trace element analysis along with pH, conductivity (Cond.), alkalinity (Alk.) and temperature values of the groundwater collected during the pre-injection period (i.e. before 2012) in well HN-01, the source of water for gas mixing at depth in the injection well HN-02, and in monitoring well HN-04 (see Supplementary Fig. 1); all data are from Alfredsson et al. (2013)[1]. In addition to values for July 2009 (Jul'09) and October 2010 (Oct'10) corresponding to microbiology-dedicated sampling periods, the average values integrates the whole set of monitoring data provided in[1]. DIC and DOC stand respectively for dissolved inorganic and organic carbon. Std dev. is for standard deviation.

**Sampling date**	**T** (°C)		**pH**		**Cond.** ($\mu S \cdot cm^{-1}$)		**O_2** ($mmole \cdot l^{-1}$)		**Alk.** ($meq. \cdot kg^{-1}$)		**DIC** ($mmole \cdot l^{-1}$)	
	**HN-01**	**HN-04**	**HN-01**	**HN-04**	**HN-01**	**HN-04**	**HN-01**	**HN-04**	**HN-01**	**HN-04**	**HN-01**	**HN-04**
**Jul'09**	23.7	33.7	9.10	9.52	236	236	0.013	0.014	1.95	1.95	1.899	1.886
**Oct'10**	28.1	30.9	9.38	9.61	295	285	0.007	0.004	1.93	2.01	1.697	1.52
Average	26	31.9	9.21	9.55	242	234	0.014	0.009	1.95	2	1.817	1.682
Std dev.	3.3	1.7	0.16	0.08	24	25	0.016	0.009	0.03	0.05	0.195	0.248

	**DOC** ($mmole \cdot l^{-1}$)		**H_2S** ($\mu mole \cdot l^{-1}$)		**SO_4** ($\mu mole \cdot l^{-1}$)		**S_{total}** ($\mu mole \cdot l^{-1}$)		**Si** ($mmole \cdot l^{-1}$)		**Na** ($mmole \cdot l^{-1}$)	
	**HN-01**	**HN-04**	**HN-01**	**HN-04**	**HN-01**	**HN-04**	**HN-01**	**HN-04**	**HN-01**	**HN-04**	**HN-01**	**HN-04**
**Jul'09**	0.040	0.021	-	0.8	0.091	0.081	0.161	0.144	0.457	1.002	1.855	2.307
**Oct'10**	0.027	0.032	-	-	0.127	0.096	0.192	0.145	0.617	1.066	2.066	2.368
Average	0.100	0.135	0.4	0.4	0.109	0.088	0.160	0.127	0.543	1.009	1.953	2.393
Std dev.	0.141	0.334	0.3	0.3	0.017	0.007	0.041	0.026	0.077	0.107	0.188	0.060

	**Cl** ($mmole \cdot l^{-1}$)		**K** ($mmole \cdot l^{-1}$)		**F** ($mmole \cdot l^{-1}$)		**Ca** ($mmole \cdot l^{-1}$)		**Mg** ($mmole \cdot l^{-1}$)		**Fe** ($\mu mole \cdot l^{-1}$)	
	**HN-01**	**HN-04**	**HN-01**	**HN-04**	**HN-01**	**HN-04**	**HN-01**	**HN-04**	**HN-01**	**HN-04**	**HN-01**	**HN-04**
**Jul'09**	0.270	0.206	0.025	0.019	0.019	0.027	0.128	0.032	0.179	0.001	0.000	0.023
**Oct'10**	0.338	0.240	0.023	0.019	0.024	0.026	0.110	0.032	0.129	0.001	0.051	0.058
Average	0.308	0.223	0.022	0.020	0.021	0.027	0.119	0.033	0.161	0.001	0.046	0.050
Std dev.	0.032	0.014	0.002	0.001	0.004	0.003	0.014	0.003	0.050	0.001	0.040	0.024

	**Al** ($\mu mole \cdot l^{-1}$)		**Sr** ($\mu mole \cdot l^{-1}$)		**Mn** ($\mu mole \cdot l^{-1}$)		**Ti** ($\mu mole \cdot l^{-1}$)		**P** ($\mu mole \cdot l^{-1}$)		**Li** ($\mu mole \cdot l^{-1}$)	
	**HN-01**	**HN-04**	**HN-01**	**HN-04**	**HN-01**	**HN-04**	**HN-01**	**HN-04**	**HN-01**	**HN-04**	**HN-01**	**HN-04**
**Jul'09**	0.535	2.664	0.209	0.013	0.078	0.002	0.003	0.004	1.439	0.304	0.000	0.141
**Oct'10**	1.031	2.897	0.158	0.013	0.119	0.000	0.003	0.001	1.250	0.328	0.000	0.000
Average	0.698	2.649	0.184	0.014	0.103	0.006	0.004	0.003	1.240	0.295	0.000	0.034
Std dev.	0.276	0.347	0.030	0.002	0.026	0.008	0.002	0.002	0.089	0.065	0.000	0.048

Supplementary Table 2 (continued)

Sampling date	Mo (µmole·l^{-1})		Br (µmole·l^{-1})		B (µmole·l^{-1})		As (µmole·l^{-1})		W (µmole·l^{-1})		Cr (µmole·l^{-1})	
	**HN-01**	**HN-04**	**HN-01**	**HN-04**	**HN-01**	**HN-04**	**HN-01**	**HN-04**	**HN-01**	**HN-04**	**HN-01**	**HN-04**
**Jul'09**	0.053	0.033	0.929	1.539	1.661	2.323	0.000	0.037	0.038	0.065	0.003	0.001
**Oct'10**	0.084	0.030	1.284	1.482	2.171	2.349	0.000	0.000	0.055	0.141	0.000	0.005
Average	0.071	0.030	1.157	1.540	1.932	2.376	0.002	0.009	0.051	0.106	0.005	0.003
Std dev.	0.009	0.006	0.255	0.121	0.354	0.113	0.005	0.017	0.008	0.044	0.006	0.003

Sampling date	Ba (µmole·l^{-1})		Sb (µmole·l^{-1})		V (µmole·l^{-1})		NO_3 (µmole·l^{-1})		N_{total} (µmole·l^{-1})		NH_4 (µmole·l^{-1})	
	**HN-01**	**HN-04**	**HN-01**	**HN-04**	**HN-04**	**HN-04**	**HN-01**	**HN-04**	**HN-01**	**HN-04**	**HN-01**	**HN-04**
**Jul'09**	0.005	0.001	0.054	0.043	0.297	0.126	0.173	2.465	-	-	-	-
**Oct'10**	0.006	0.001	0.037	0.040	0.161	0.136	-	-	-	-	-	-
Average	0.006	0.001	0.025	0.043	0.217	0.146	0.239	0.463	4.593	3.627	1.120	1.32
Std dev.	0.001	0.001	0.020	0.022	0.039	0.023	0.241	0.716		0.182	0.272	0.464

Supplementary Table 3. Elemental concentrations in the groundwater from control well HN-01 and monitoring well HN-04 filtered for microbial ecology analysis (see also Supplementary Fig. 2 for temporal evolution of some targeted elements)**.** It is noticeable that for the same sampling period, elemental concentrations in HN-01 groundwater are for some elements largely higher than the ones in HN-04 groundwater. As HN-01 groundwater were co-injected with the gases in the aquifer, they hence could have chemically influenced the injection and monitoring wells, with likely consequences on the microbial ecology. Those are nonetheless difficult to assess as masked by the natural variability induced by the aquifer dynamics (Fig. 4). Accordingly, only values significantly differing from HN-01 background values and assignable to the interactions between the host rock and the fast flowing fraction of the acidifying CO_2 injectat were here considered to discuss the microbial reactivity in the aquifer as evidenced in HN-04 monitoring well (in bold). Relative standard deviations are indicated in %. bd stands for below detection. Full geochemical monitoring during the injection can be found in Snæbjörnsdóttir et al. (2017)[6].

	HN-01				HN-04				
(ppb)	Feb'12	Mar'12	May12	Jul'12	Feb'12	Mar'12	May12	Jul'12	Jun'13
Li	0.186 ±2.7	1.702 ±1.2	1.839 ±0.7	1.571 ±0.6	0.368 ±1.6	1.31 ±2.2	1.752 ±2.2	1.893 ±1.1	0.812 ±1.8
B	29.10 ±1.0	690.32 ±1.4	777.65 ±1.6	649.1 ±2.0	26.58 ±1.1	384.61 ±1.8	548.36 ±0.7	636.73 ±2.8	77.34 ±1.6
Na	33751.64 ±0.6	32973.05 ±0.9	33640.85 ±0.8	32753.38 ±0.1	36895.45 ±0.3	40349.88 ±0.9	42179.21 ±0.5	41176.06 ±0.6	46081.58 ±1.4
**Mg**	**3356.74 ±0.5**	**3554.94 ±0.5**	**3434.81 ±0.9**	**3650.05 ±0.3**	**635.66 ±0.2**	**1639.87 ±0.3**	**402.26 ±0.5**	**1190.23 ±0.4**	**804.47 ±0.2**
Al	4.91 ±16.9	13.81 ±3.7	6.45 ±1.7	16.26 ±2.3	1.09 ±13.1	18.57 ±2.6	5.95 ±15.1	6.64 ±5.3	10.03 ±3.7
K	922.2 ±0.6	798.68 ±0.5	805.5 ±0.3	819.68 ±0.4	741.01 ±0.8	692.44 ±0.6	730.99 ±0.6	689.95 ±0.1	881.5 ±0.4
**Ca**	**668.35 ±0.9**	**676.16 ±0.4**	**657.18 ±0.9**	**686.88 ±0.2**	**573.72 ±0.5**	**1700.00 ±0.3**	**500.16 ±0.5**	**1264.27 ±0.4**	**842.06 ±0.4**
V	10.12 ±1.0	7.61 ±1.9	8.23 ±0.1	8.9 ±0.4	2.9 ±2.1	0.98 ±3.6	1.89 ±0.7	1.65 ±0.8	2.10 ±2.1
Cr	0.246 ±5.3	0.136 ±8.3	0.165 ±4.7	0.176 ±7.4	0.194 ±4.2	0.14 ±7.0	0.148 ±2.9	0.208 ±4.1	0.156 ±5.4
**Mn**	**0.02 ±3.3**	**0.95 ±2.0**	**0.3 ±2.7**	**0.7 ±4.8**	**0.44 ±8.9**	**26.63 ±0.8**	**0.25 ±3.8**	**8.93 ±1.4**	**1.87 ±1.1**
**Fe**	**2.95 ±6.0**	**1.52 ±15.9**	**bd**	**3.23 ±6.0**	**bd**	**1170.97 ±0.8**	**6.22 ±12.8**	**15.43 ±1.5**	**1.7 ±16.5**
**Ni**	**0.0161 ±15.6**	**bd**	**bd**	**bd**	**0.0155 ±25.1**	**0.4512 ±1.6**	**0.0215 ±27.0**	**0.0386 ±11.8**	**0.04 ±25.9**
Cu	0.3476 ±2.9	0.9438 ±2.0	0.0544 ±19.7	0.1494 ±3.3	0.1935 ±3.5	0.2732 ±3.2	0.1817 ±2.5	0.1828 ±4.5	25.4904 ±2.6
**Zn**	**0.67 ±4.1**	**5.69 ±3.6**	**4.62 ±3.2**	**6.02 ±0.6**	**0.14 ±12.3**	**72.77 ±0.6**	**2.7 ±3.3**	**12.63 ±1.1**	**8.27 ±2.5**
Rb	0.838 ±0.8	0.597 ±0.5	0.636 ±1.9	0.635 ±1.6	1.087 ±0.6	0.841 ±0.7	0.881 ±1.0	0.891 ±0.5	1.307 ±0.9
Sr	14.462 ±0.8	15.138 ±0.6	14.643 ±1.2	15.148 ±0.2	3.277 ±0.6	6.527 ±0.2	2.629 ±1.2	6.373 ±0.3	6.001 ±0.3
**Zr**	**0.0164 ±2.0**	**0.0125 ±5.2**	**0.012 ±15.0**	**0.0286 ±4.6**	**0.0095 ±4.7**	**0.1321 ±0.7**	**0.0162 ±13.0**	**0.0544 ±2.3**	**0.0047 ±20.4**
Mo	2.88 ±1.0	3.01 ±1.1	3.48 ±0.3	3.43 ±1.1	1.78 ±1.0	1.96 ±1.4	2.45 ±1.7	2.11 ±3.0	1.51 ±1.7
Cs	0.0066 ±8.0	0.014 ±3.6	0.0165 ±4.5	0.0149 ±5.9	0.009 ±1.0	0.0099 ±9.2	0.0146 ±1.7	0.0176 ±4.2	0.0111 ±0.7
Ba	33.91 ±0.3	95.71 ±0.4	106.67 ±1.0	97.32 ±1.7	58.95 ±0.4	77.84 ±0.1	60.82 ±1.6	115.22 ±0.7	62.23 ±1.1
W	0.7863 ±1.6	0.8151 ±1.1	0.8713 ±0.9	0.8516 ±1.5	1.1432 ±1.3	1.0516 ±0.6	1.5555 ±0.9	1.3109 ±1.9	1.1883 ±1.6
Hg	0.2766 ±5.7	0.6529 ±7.2	0.6440 ±3.7	0.5208 ±2.9	1.0879 ±3.8	0.3200 ±2.8	0.2222 ±7.7	0.9042 ±5.4	0.3802 ±3.6
**Pb**	**0.0032 ±19.4**	**0.0125 ±2.1**	**0.0124 ±7.7**	**0.0213 ±3.5**	**0.0074 ±1.9**	**0.1007 ±3.0**	**0.0184 ±6.0**	**0.1174 ±1.6**	**0.0235 ±1.6**
U	0.0243 ±3.3	0.0297 ±1.7	0.0301 ±2.0	0.0286 ±3.0	0.0082 ±2.2	0.0445 ±2.2	0.0159 ±0.9	0.0464 ±2.0	0.0188 ±0.9

Supplementary Table 4. Primers and thermal conditions used for PCR amplifications of functional genes related to inorganic carbon assimilation and aromatic carbon degradation. Successful amplifications were obtained when targeting (1) *cbbL* and *cbbM* genes, respectively encoding form I and form II ribulose-1,5-bisphosphate carboxylase/oxygenase (RuBisCO), a key enzyme for autotrophic CO_2 fixation, (2) the genes coding for the largest subunit of multicomponent phenol hydroxylases (LmPHs) involved in the degradation of phenolic compounds. Although the Calvin-Benson-Bassham (CBB) cycle was the only pathway of CO_2 fixation detected by metagenomic analyses (Fig. 6 and Supplementary Table 7), we also tried to selectively amplify, the other genes involved in autotrophic C-fixation. Those included (1) *accA* encoding the acetyl-CoA carboxylase alpha subunit involved in the 3-hydroxypropionate/4-hydroxybutyrate (HP/HB or 3HP/4HB) cycle, (2) *aclB* encoding Beta ATP citrate lyase involved in the reductive Tricarboxylic Acid cycle, (3) *pcs* gene encoding the propionyl-CoA synthase along with *mcrA* as part of the genes involved in the 3-Hydroxypropionate (3HP) bicycle, (4) *acs* encoding acetyl-CoA synthetase for the reductive acetyl CoA pathway.

Primers	Sequence (5' – 3')	Thermal conditions	References
cbbL			
cbbL F<br>cbbL R<br>(711 bp)	GACTTCACCAAAGACGACGA<br>TCGAACTTGATTTCTTTCCA	95°C, 4 min, 1 cycle<br>94°C for 45 s, 53°C for 45 s,<br>72°C for 1 min, 35 cycles<br>72°C for 10 min, 1 cycle	[23]
cbbM			
cbbM343F<br>cbbM1226R<br>(800 to 900 bp)	GGYAAYAACCARGGYATGGG<br>CGYARBGCRTTCATRCCRCC	95°C, 2 min, 1 cycle<br>95°C for 1 min, 50°C for 2 min,<br>72°C for 3 min, 30 cycles<br>72°C for 10 min, 1 cycle	[24]
**LmPHs encoding genes**			
pheUf<br>pheUr<br>(700 bp)	CCAGGSBGARAARGAGARGAARCT<br>CGGWARCCGCGCCAGAACCA	94°C, 5 min, 1 cycle<br>94°C for 1 min, 58°C for 1 min,<br>72°C for 1 min, 5 cycles<br>94°C for 1 min, 57°C for 1 min,<br>72°C for 1 min, 5 cycles<br>94°C for 1 min, 56°C for 1 min,<br>72°C for 1 min, 25 cycles<br>72°C for 10 min, 1 cycle	[25]
PHE-F<br>PHE-R<br>(700 bp)	GAYCCBTTYCGYHTRACCATGGA<br>GGCARCATGTARTCCWKCATCAT	95°C, 5 min, 1 cycle<br>95°C for 45 s, 52°C for 45 s,<br>72°C for 1 min, 35 cycles<br>72°C for 8 min, 1 cycle	[26]

f/F, forward primer ; r/R, reverse primer

Supplementary Table 5. Mantel correlations of groundwater phylogenetic diversity (unweighted Unifrac[27]; Figs. 2 and 3) **from control well HN-01 and monitoring well HN-04 and associated environmental parameters** (Supplementary Tables 2 and 3). Mantel test was performed with 9,999 permutations, based on Pearson's product-moment. p-values were obtained with alpha=0.05. DIC and DOC stand for dissolved inorganic and organic carbon, respectively. n.a. stands for not applicable.

Parameters	HN-01 & HN-04		HN-01		HN-04	
	r-statistic	p-value	r-statistic	p-value	r-statistic	p-value
Li	0.0604	0.1166	0.3004	0.0217*	0.1552	1.24E-001
B	0.0443	0.2219	0.2321	0.0441*	0.2171	0.053
Na	0.2044	0.0324*	**0.6660**	0.0016**	0.2738	7.34E-002
Mg	0.4755	0.0001***	**0.6910**	0.0001***	**0.6598**	0.0001***
Al	0.1638	0.1011	0.2186	0.0934	0.2588	8.46E-002
K	0.3409	0.0027**	0.4861	0.0007***	0.3806	0.0166*
Ca	0.0177	0.3754	**0.7496**	0.0003***	0.4089	0.0065**
V	0.4944	0.0002***	0.5868	0.0075**	0.3830	0.0156*
Cr	(-)0.0391	0.5738	0.2706	0.0705	0.0670	0.3273
Mn	0.6763	0.0004***	**0.6427**	0.0021**	**0.7854**	0.0061**
Fe	0.6980	0.0001***	0.0786	0.2069	**0.8355**	0.001***
Sr	0.4818	0.0001***	**0.6902**	0.0001***	0.4183	0.0016**
Mo	0.0902	0.2119	0.5243	0.0166*	0.3093	0.0225*
Ba	0.0994	0.0714	0.4956	0.0019**	0.2291	6.54E-002
W	0.1257	0.1677	**0.6695**	0.0013**	0.1451	0.1868
T	0.0696	0.2243	0.0702	0.189	0.5498	0.0009***
pH	0.7326	0.0001***	**0.7553**	0.0003***	**0.8320**	0.0004***
Conductivity	0.4842	0.0001***	0.5617	0.0068**	0.4646	0.0022**
DOC	n.a.	n.a.	n.a.	n.a.	**0.8344**	0.0011*
DIC	n.a.	n.a.	n.a.	n.a.	**0.8541**	0.0001***
Na_fluorescein	n.a.	n.a.	n.a.	n.a.	0.1730	0.1448

*p<0.05 **p<0.01 ***p<0.001

Supplementary Table 6. Closest cultivated and environmental relatives of the bacterial OTUs retrieved by cloning and Sanger sequencing from HN-04 groundwater in March and May'12.Sequences from March'12 and May'12 are shown in light gray and white, respectively. Most abundant OTUs are shown in bold.

Sequence #ID	Accession number	Number of clones	Taxonomic affiliation (SINA)[28]	Closest environmental clone	%	Closest cultivated bacteria	%
1_70	KX276759	2	Chlorobi	FR667795 - iron snow from acidic coal mine- lake	97		
1_10	KX276726	1	Chlorobi	AB924432 - deep groundwater	96		
1_8	KX276730	1	Chlorobi	DQ463733 - lake Tanganyika anoxic hypolimnion	96		
1_4	KX276727	1	Chlorobi	AY604049 - Chuniespoort group dolomite at 896 meters depth	97	NR_074796 *Melioribacter roseus* P3M-2	94
1_73	KX276757	1	Chloroflexi; Anaerolineae; Anaerolineales; Anaerolineaceae	EU266865 - tar-oil contaminated aquifer sediments	99		
1_2	KX276733	1	Firmicutes; Clostridia; Clostridia Incertae Sedis; Unknown Family; Candidatus *Desulforudis*	AM777962 - alkaline groundwater associated with serpentinization	99	NR_075067 Candidatus *Desulforudis audaxviator* MP104C	99
**1_15**	**KX276721**	**14**	**Firmicutes; Clostridia; Clostridiales; Peptococcaceae; *Desulfotomaculum***	**KF939360 - deep geothermal aquifer associated with fractured Paleozoic carbonate rock**	**99**	**KF591692 *Desulfotomaculum profondi***	**99**
1_12	KX276724	1	Firmicutes; Clostridia; Clostridiales; Syntrophomonadaceae; *Dethiobacter*	DQ088768 - 0.7 to 1.4 km section of continental crust	96	NR_044205 *Dethiobacter alkaliphilus* AHT 1	91
1_50	KX276750	1	Nitrospirae; Nitrospira; Nitrospirales; Nitrospiraceae; Nitrospira	FJ793169 - Tao Dam hot spring	99	CP011801 *Nitrospira moscoviensis* strain NSP M-1	98
1_1	KX276734	2	Nitrospirae; Nitrospira; Nitrospirales; Nitrospiraceae	KJ650735 - Selebi-Phikwe tailing dump	97		
1_7	KX276731	3	Nitrospirae	AM039540 - subsurface thermal spring	98		
1_67	KX276762	1	Proteobacteria; Alphaproteobacteria; Sphingomonadales; Sphingomonadaceae	KM851964 - drinking water biofilm	99	KM852537 *Rhizorhabdus* sp. YGR-17	99
1_64	KX276765	2	Proteobacteria; Betaproteobacteria	JQ655312 - drinking waters	99		
1_28	KX276737	1	Proteobacteria; Betaproteobacteria; Burkholderiales; Comamonadaceae	AB780355 - sediment of Lake Jusan	98		
1_6	KX276729	1	Proteobacteria; Betaproteobacteria; Burkholderiales; Comamonadaceae	KT698872 - coal-bed water	99	DQ413156 *Hydrogenophaga* sp. EMB 85	99
1_65	KX276764	1	Proteobacteria; Betaproteobacteria; Burkholderiales; Comamonadaceae; *Hydrogenophaga*	FJ037628 - biofilms growing on the tunnel walls of the ONKALO tunnel in Olkiluoto	99	DQ413156 *Hydrogenophaga* sp. EMB 85	98

Supplementary Table 6 (continued)

Sequence #ID	Accession number	Number of clones	Taxonomic affiliation (SINA)[28]	Closest environmental clone	%	Closest cultivated bacteria	%
12a-7c	KU685485	1	Proteobacteria; Betaproteobacteria; Burkholderiales; Comamonadaceae; *Hydrogenophaga*	AB924427 - deep groundwater	99	DQ413146 *Hydrogenophaga* sp. EMB 33	98
12a-10c	KU685488	2	Proteobacteria; Betaproteobacteria	AB924418 - deep groundwater	96		
1_13	KX276723	1	Proteobacteria; Betaproteobacteria; Hydrogenophilales; Hydrogenophilaceae; *Sulfuricella*	GQ388960 - drinking water distribution system during serious red water outbreak	99	NR_121695 *Sulfuricella denitrificans* skB26	99
1_17	KX276718	3	Proteobacteria; Betaproteobacteria; Hydrogenophilales; Hydrogenophilaceae; *Thiobacillus*	DQ088746 - 0.7 to 1.4 km section of the continental crust	100	CP000116 *Thiobacillus denitrificans* ATCC 25259	99
1_60	KX276747	1	Proteobacteria; Betaproteobacteria; Hydrogenophilales; Hydrogenophilaceae	DQ230972 - subsurface water	99		
1_51	KX276749	1	Proteobacteria; Betaproteobacteria; Hydrogenophilales; Hydrogenophilaceae	EU746750 - drinking water system	99		
12e-30c	KU685504	1	Proteobacteria; Betaproteobacteria; Nitrosomonadales; Gallionellales; Gallionellaceae	EU266836 - tar-oil contaminated aquifer sediments	96	DQ839562 Candidatus *Nitrotoga arctica*	96
12a-15c	KU685490	1	Proteobacteria; Betaproteobacteria; Nitrosomonadales; Gallionellales; Gallionellaceae	EU266836 - tar-oil contaminated aquifer sediments	98	DQ839562 Candidatus *Nitrotoga arctica*	96
12a-20C	KU685492	6	Proteobacteria; Betaproteobacteria; Nitrosomonadales; Gallionellales; Gallionellaceae	EU266836 - tar-oil contaminated aquifer sediments	96	DQ839562 Candidatus *Nitrotoga arctica*	95
12a-23c	KU685493	1	Proteobacteria; Betaproteobacteria; Nitrosomonadales; Gallionellales; Gallionellaceae	EU266836 - tar-oil contaminated aquifer sediments	96	DQ839562 Candidatus *Nitrotoga arctica*	95
12a-48c	KU685501	1	Proteobacteria; Betaproteobacteria; Nitrosomonadales; Gallionellales; Gallionellaceae	EF562112 - Henderson molybdenum mine	97	CP001965 *Sideroxydans lithotrophicus* strain ES-1	96
12a-28c	KU685495	4	Proteobacteria; Betaproteobacteria; Nitrosomonadales; Gallionellales; Gallionellaceae; *Ferriphasellus*	Q278891 - chlorinated aliphatic hydrocarbon in groundwater	99	NR_114334 *Ferriphaselus amnicola*	99
1_61	KX276768	1	Proteobacteria; Betaproteobacteria; Nitrosomonadales; Gallionellales; Gallionellaceae	HM572475 - riverine rock	97	NR_074658 *Gallionella capsiferriformans* ES-2	96
12a-1c	KU685482	1	Proteobacteria; Betaproteobacteria; Nitrosomonadales; Gallionellales; Gallionellaceae	AF351237 - coal-tar-waste-contaminated aquifer waters	96	DQ386859 *Sideroxydans lithotrophicus* strain LD-1	96

Supplementary Table 6 (continued)

Sequence #ID	Accession number	Number of clones	Taxonomic affiliation (SINA)[28]	Closest environmental clone	%	Closest cultivated bacteria	%
**12a-42c**	**KU685499**	**12**	**Proteobacteria; Betaproteobacteria; Nitrosomonadales; Gallionellales; Gallionellaceae**	**EF562112 - Henderson molybdenum mine**	**95**	**DQ386859 *Sideroxydans lithotrophicus* strain LD-1**	**94**
12a-44c	KU685500	2	Proteobacteria; Betaproteobacteria; Nitrosomonadales; Gallionellales. Gallionellaceae; *Sideroxydans*	GQ389143 - drinking water distribution system during serious red water outbreak	98	DQ386859 *Sideroxydans lithotrophicus* strain LD-1	97
12a-5c	KU685483	1	Proteobacteria; Betaproteobacteria; Nitrosomonadales; Gallionellaceae	GQ389143 - drinking water distribution system during serious red water outbreak	97	DQ386859 *Sideroxydans lithotrophicus* strain LD-1	96
12e-21c	KU685502	2	Proteobacteria; Betaproteobacteria; Nitrosomonadales; Gallionellaceae	GQ389141 - drinking water distribution system during serious red water outbreak	96	CP001965 *Sideroxydans lithotrophicus* strain ES-1	96
12a-38c	KU685498	3	Proteobacteria; Betaproteobacteria; Rhodocyclales; Rhodocyclaceae	AF351237 - coal tar waste-contaminated groundwater	96	DQ386859 *Sideroxydans lithotrophicus* strain LD-1	96
12a-33c	KU685497	1	Proteobacteria; Betaproteobacteria; Rhodocyclales; Rhodocyclaceae	LC122006 - groundwater, Tono uranium mine	95	DQ386859 *Sideroxydans lithotrophicus* strain LD-1	95
12a-27c	KU685494	1	Proteobacteria; Betaproteobacteria; Rhodocyclales; Rhodocyclaceae	LC122006 - groundwater, Tono uranium mine	99	DQ839562 Candidatus *Nitrotoga arctica*	94
12a-18c	KU685491	1	Proteobacteria; Betaproteobacteria; Rhodocyclales; Rhodocyclaceae	LC122006 - groundwater, Tono uranium mine	96	JQ751310 *Zoogloea* sp. HJ1	94
12e-23c	KU685487	1	Proteobacteria; Betaproteobacteria; Rhodocyclales; Rhodocyclaceae	LC122006 - groundwater, Tono uranium mine	97	DQ839562 Candidatus *Nitrotoga arctica*	95
**12e-27c**	**KU685503**	**26**	**Proteobacteria; Betaproteobacteria; Rhodocyclales; Rhodocyclaceae**	**LC122006 - groundwater, Tono uranium mine**	**97**	**DQ839562 Candidatus *Nitrotoga arctica***	**94**
12a-13c	KU685489	2	Proteobacteria; Betaproteobacteria; Rhodocyclales; Rhodocyclaceae	LC122006 - groundwater, Tono uranium mine	96	CP001965 *Sideroxydans lithotrophicus* strain ES-1	94
12a-6c	KU685484	1	Proteobacteria; Betaproteobacteria; Rhodocyclales; Rhodocyclaceae	LC122006 - groundwater, Tono uranium mine	95	CP001965 *Sideroxydans lithotrophicus* strain ES-1	94
1_34	KX276745	3	Proteobacteria; Betaproteobacteria; Rhodocyclales; Rhodocyclaceae	LC122006 - groundwater, Tono uranium mine	100		
1_72	KX276758	1	Proteobacteria; Betaproteobacteria; Rhodocyclales; Rhodocyclaceae	FJ429710 - groundwater	97		
**12a-9c**	**KU685486**	**10**	**Proteobacteria; Betaproteobacteria; Rhodocyclales; Rhodocyclaceae**	**AB924427 - deep groundwater**	**99**	**AP012547 *Sulfuritalea hydrogenivorans* sk43H**	**95**

Supplementary Table 6 (continued)

Sequence #ID	Accession number	Number of clones	Taxonomic affiliation (SINA)[28]	Closest environmental clone	%	Closest cultivated bacteria	%
1_62	KX276767	2	Proteobacteria; Betaproteobacteria; Rhodocyclales; Rhodocyclaceae	LC122006 - groundwater, Tono uranium mine	97		
1_49	KX276751	5	Proteobacteria; Betaproteobacteria; Rhodocyclales; Rhodocyclaceae	KF836328 - springs and wells fed by a deep fractured rock aquifer in the Mojave Desert	97		
1_32	KX276746	1	OP11 Microgenomates	FJ710737 - anaerobic ammonium oxidation reactor	97		

Supplementary Table 7. Metabolic capability of the bacterial community sampled in HN-04 monitoring well in March and May 2012 (Mar'12 and May'12, respectively) as inferred by metagenomic analyses. For all encoded proteins, identity number from COGs analysis, name of the encoding gene and normalized number of retrieved sequences are reported.

**CO_2 fixation**			**Mar'12**	**May'12**
**Reductive pentose phosphate or Calvin–Benson–Bassham (CBB) cycle**				
Phosphoribulokinase	COG3954	*prkB*	0.992298578	0.646772025
Ribulose 1,5-bisphosphate carboxylase, large subunit, or a RuBisCO-like protein	COG1850	*rbcL*	1.741478308	1.018975696
Ribulose bisphosphate carboxylase small subunit	COG4451	*rbcS*	0.188662049	0.161073493
**Degradation of aromatic compounds**			**Mar'12**	**May'12**
**Anaerobic degradation of aromatic compounds**				
Benzoyl-CoA reductase/2-hydroxyglutaryl-CoA dehydratase subunit, *bcrC*/*badD*/*hgdB*	COG1775	*hgdB*	1.13630149	0.66114474
**Aerobic degradation of aromatic compounds**				
Aromatic ring hydroxylase	COG2368	*yoaI*	0.08590047	0.19997894
Aromatic ring-opening dioxygenase, catalytic subunit, *ligB* family	COG3384	*ligB*	1.33749544	0.33131578
Dienelactone hydrolase	COG0412	DLH	1.26731681	0.85864562
Catechol 2,3-dioxygenase or other lactoylglutathione lyase family enzyme	COG0346	*gloA*	1.56967736	1.27248059
Ferredoxin subunit of nitrite reductase or a ring-hydroxylating dioxygenase	COG2146	*nirD*	0.51016223	0.4334116
Phenylpropionate dioxygenase or related ring-hydroxylating dioxygenase, large terminal subunit	COG4638	*hcaE*	1.04151476	0.82742214
Predicted class III extradiol dioxygenase/MEMO1 family	COG1355	*mho1*	0.50879511	0.67601306
Predicted oxidoreductase (related to aryl-alcohol dehydrogenase)	COG0667	*tas*	1.20032811	1.00286835
2-polyprenyl-6-methoxyphenol hydroxylase and related FAD-dependent oxidoreductases	COG0654	*ubiH*	3.78941852	1.64121499
**Acetyl CoA pathway/fermentation**			**Mar'12**	**May'12**
**Oxidative acetyl coenzyme A (CoA) pathway**				
CO dehydrogenase/acetyl-CoA synthase alpha subunit	COG1152	*cdhA*	0.046254101	0.32512065

Supplementary Table 7 (continued)

CO dehydrogenase/acetyl-CoA synthase beta subunit	COG1614	*cdhC*	0.046026249	0.287206428
CO dehydrogenase/acetyl-CoA synthase delta subunit (corrinoid Fe-S protein)	COG2069	*cdhD*	0.043519869	0.175446205
CO dehydrogenase/acetyl-CoA synthase epsilon subunit	COG1880	*cdhB*	0.00250638	0.004708302
CO dehydrogenase/acetyl-CoA synthase gamma subunit (corrinoid Fe-S protein)	COG1456	*cdhE*	0.042380605	0.280020072
**Fermentation**				
Pyruvate:ferredoxin oxidoreductase or related 2-oxoacid:ferredoxin oxidoreductase, alpha subunit	COG0674	*porA*	4.9562522	4.8666497
Pyruvate:ferredoxin oxidoreductase or related 2-oxoacid:ferredoxin oxidoreductase, beta subunit	COG1013	*porB*	1.6601348	1.5703426
Pyruvate:ferredoxin oxidoreductase or related 2-oxoacid:ferredoxin oxidoreductase, gamma subunit	COG1014	*porG*	3.9719285	1.9636097
Pyruvate:ferredoxin oxidoreductase or related 2-oxoacid:ferredoxin oxidoreductase, delta subunit	COG1144	*porD*	0.0913689	0.0894577
Phosphotransacetylase	COG0280	*pta*	4.04985417	2.13087841
Acetate kinase	COG0282	*ackA*	0.93305687	0.87970908

**CO oxidation**			**Mar'12**	**May'12**
**Aerobic carbon monoxide dehydrogenase (CODH) = CO oxidation***				
Aerobic-type carbon monoxide dehydrogenase, small subunit, *coxS*/*cutS* family	COG2080	*coxS*	0.174079475	0.459926774
Carbon monoxide dehydrogenase, subunit G	COG3427	*coxG*	0.021418155	0.030480061

**S metabolisms**			**Mar'12**	**May'12**
Adenylyl sulfate reductase	COG1053	*aprA*	3.03773241	3.5830179
Adenylyl sulfate reductase	COG1146	*aprB*	0.70201422	1.14238277
Sulfite reductase,dissimilatory type	COG2221	*dsrAB*	1.17366934	1.82682121
Sulfur oxidation protein *soxZ*	COG2033 COG5501	*soxZ*	0.53522603	0.50775321
Sulfur oxidation protein *soxY* related AACIE arm protein	COG5501	*soxY*	0.50446591	0.42126914
Sulfite oxidase EC 1.8.3.1	COG2041	*yedY*	0.46778163	0.70277604

Supplementary Table 7 (continued)

Sulfite dehydrogenase	COG3258<br>COG4654	*cytC*	1.36210354	0.70500629
**Fe metabolisms**			**Mar’12**	**May’12**
MR-1 decaheme cytochrome *mtrA*	COG3005	*mtrA*	0.82710536	0.97907903
Decaheme cytochrome	COG3637?	*mtrB*	0.22489063	0.12266366
Hemerytrin	COG2703	*hr*	1.2096701	0.4562097
Truncated hemoglobin	COG2346	*hbN*	0.3392727	0.2081565
Cbb3-type cytochrome oxidase, subunit 1	COG3278	*ccoN*	1.5211447	1.2023517
Cbb3-type cytochrome oxidase, subunit 3	COG4736	*ccoQ*	0.0581024	0.0748372
Cbb3-type cytochrome oxidase, cytochrome c subunit	COG2993	*ccoO*	0.8059151	0.7912426
Cytochrome b subunit of the bc complex	COG1290	*qcrB*	1.36460991	1.27347181
Cytochrome c1	COG2857	*cyt1*	0.63274699	0.35287485
Cytochrome c551/c552	COG4654	*cytC552*	0.77037003	0.29364936
NADH dehydrogenase/NADH:ubiquinone oxidoreductase 75 kD subunit (chain G)	COG1034	*nuoG*	2.34050309	1.46378634
**N metabolisms**			**Mar’12**	**May’12**
Copper containing nitrite reductase	COG2132	*nirK*	0.63912687	1.71580441
Cytochrome cd1 containing nitrite reductase	COG2010	*nirS*	6.61365293	4.45578842
NAD(P)H-nitrite reductase, large subunit	COG1251	*nirB*	3.28700328	1.49847909
Nitrous oxide reductase	COG4263	*nosZ*	1.18597339	0.8437773
Nitrogenase subunit	COG1348	*nifH*	0.59355633	0.25623076
Nitrogenase subunit	COG2710	*nifD*	3.55495808	1.56117385

Supplementary Table 8. Occurrences of homologs of the *cyc2* gene involved in Fe-oxidation and present in *Sideroxydans lithotrophicus* ES-1 and *Mariprofundus ferrooxydans* PV-1[29,30] in the metagenomic analyses of the bacterial community sampled in HN-04 monitoring well in March and May 2012 (Mar'12 and May'12, respectively). Number of hits matching respectively ADE10507/Slit_0265 and AKN78226.1/Cyc2PV-1 for *S. lithotrophicus* ES-1 and *M. ferrooxydans* PV-1, are reported for both sampling periods along with the normalized number of retrieved sequences.

***cyc2* gene homolog**	**Hits in Mar'12**	**Hits in May'12**	**Normalized number of sequences for Mar'12**	**Normalized number of sequences for May'12**
ADE10507:Slit_0265	2495	129	0.5684	0.0319
AKN78226.1:Cyc2PV-1	341	85	0.0777	0.0210

Supplementary Table 9. Primers and thermal conditions used for real-time PCR quantification. The target gene was the 16S-rRNA gene, except for *amoA* functional gene quantifications.

Primers	Sequence (5' – 3')	Thermal conditions	References
**Eubacteria**<br>341F<br>534	CCT ACG GGA GGC AGC AG<br>ATT ACC GCG GCT GCT GGC A	95°C, 10 min, 1 cycle<br>95°C for 15 s, 60°C for 1 min, 35 cycles<br>95°C for 15 s, 60 to 95°C, 1 cycle	[31,32]
**Betaproteobacteria**<br>Beta 359f<br>Beta 682r	GGG GAA TTT TGG ACA ATG GG<br>ACG CAT TTC ACT GCT ACA CG	95°C, 10 min, 1 cycle<br>95°C for 15 s, 60°C for 1 min, 35 cycles<br>95°C for 15 s, 60 to 95°C, 1 cycle	[33]
**Archaea**<br>Parch519f<br>Parch915r	CMG CCG CGG TAA<br>GTG CTC CCC CGC CAA TTC CT	95°C for 15 s, 60°C for 1 min, 35 cycles<br>95°C for 15 s, 60 to 95°C, 1 cycle	[34,35]
**Crenarchaeota /Thaumarchaeota**<br>Crenar771F<br>Crenar957R	ACGGTGAGGGATGAAAGCT<br>CGGCGTTGACTCCAATTG	95°C, 10 min, 1 cycle<br>95°C for 15 s, 55°C for 30s, 72°C for 1 min, 35 cycles<br>95°C for 15 s, 60 to 95°C, 1 cycle	[36]
***amoA* Archaea**<br>CrenamoA23F<br>CrenamoA616r	ATG GTC TGG CTW AGA CG<br>GCCATC CAT CTG TATGTCCA	95°C, 10 min, 1 cycle<br>95°C for 15 s, 55°C for 30s, 72°C for 1 min, 35 cycles<br>95°C for 15 s, 60 to 95°C, 1 cycle	[37]

f/F, forward primer ; r/R, reverse primer

Supplementary References

1. Alfredsson, H. A. *et al.* The geology and water chemistry of the Hellisheidi, SW-Iceland carbon storage site. *Int. J. Greenh. Gas Control* **12**, 399-418 (2013).
2. Aradóttir, E. S. P., Sonnenthal, E. L., Björnsson, G. & Jónsson, H. Multidimensional reactive transport modeling of CO_2 mineral sequestration in basalts at the Hellisheidi geothermal field, Iceland. *Int. J. Greenh. Gas Control* **9**, 24-40 (2012).
3. Gislason, S. R. & Oelkers, E. H. Carbon storage in basalt. *Science* **344**, 373-374 (2014).
4. Matter, J. M. *et al.* Permanent carbon dioxide storage into basalt: the CarbFix pilot project, Iceland. *Energy Procedia* **1**, 3641–3646 (2009).
5. Sigfusson, B. *et al.* Solving the carbon-dioxide buoyancy challenge: The design and field testing of a dissolved CO_2 injection system. *Int. J. Greenh. Gas Control* **37**, 213-219 (2015).
6. Snæbjörnsdóttir, S. Ó. *et al.* CarbFix: The chemistry and saturation states of subsurface fluids during the in situ mineralisation of CO_2 and H_2S at the CarbFix site in SW-Iceland. *Int. J. Greenh. Gas Control* **58**, 87-102 (2017).
7. Matter, J. M. *et al.* Monitoring permanent CO_2 storage by in situ mineral carbonation using a reactive tracer technique. *Energy Procedia* **63**, 4180-4185 (2014).
8. Rezvani Khalilabad, M., Axelsson, G. & Gislason, S. R. Rapid carbon mineralization for permanent disposal of anthropogenic carbon dioxide emissions. *Mineral. Mag.* **72**, 121-125 (2008).
9. Kellerman, A. M., Dittmar, T., Kothawala, D. N. & Tranvik, L. J. Chemodiversity of dissolved organic matter in lakes driven by climate and hydrology. *Nat. Commun.* **5**, 3804 (2013).
10. Einsiedl, F. *et al*. Rapid biotic molecular transformation of fulvic acids in a karst aquifer. *Geochim. Cosmochim. Acta* **71**, 5474-5482 (2007).
11. Dvorski, S. E. -M. *et al.* Geochemistry of Dissolved Organic Matter in a spatially highly resolved groundwater petroleum hydrocarbon plume cross-section. *Environ. Sci. Technol.* **50**, 5536-5546 (2016).
12. Rainer, U. *et al.* Water droplets in oil are microhabitats for microbial life. *Science* **345**, 673-676 (2014).
13. Jukes, T. H. & Cantor, C. R. Evolution of protein molecules. in *Mammalian Protein Metabolism* (ed Munro, H. N.) 21-132 (Academic Press, 1969).
14. Tamura, K., Stecher, G., Peterson, D., Filipski, A. & Kumar, S. MEGA6: Molecular Evolutionary Genetics Analysis version 6.0. Mol. *Biol. Evol.* **30**, 2725-2729 (2013).
15. Ludwig, W. *et al.* ARB: a software environment for sequence data. *Nucleic Acids Res.* **32**, 1363-1371 (2004).
16. Pester, M., Schleper, C. & Wagner, M. The *Thaumarchaeota*: an emerging view of their phylogeny and ecophysiology. *Curr. Opin. Microbiol.* **14**, 300-306 (2011).
17. Kan, J. *et al.* Archaea in Yellowstone lake. *ISME J.* **5**, 1784-1795 (2011).
18. Takai, K., Komatsu, T., Inagaki, F. & Horikoshi, K. Distribution of Archaea in a black smoker chimney structure. *Appl. Environ. Microbiol.* **67**, 3618-3629 (2001).
19. Karl, D. M., McMurtry, G. M., Malahoff, A. & Garcia, M. O. Loihi Seamount, Hawaii: a mid-plate volcano with a distinctive hydrothermal system. *Nature* **335**, 533-535 (1988).
20. Moyer, C. L., Dobbs, F. C. & Karl, D. M. Phylogenetic diversity of the bacterial community from a microbial mat at an active, hydrothermal vent system, Loihi seamount, Hawaii. *Appl. Environ. Microbiol.* **61**, 1555-1562 (1995).
21. Emerson, D. & Moyer, C. L. Neutrophilic iron-oxidizing bacteria are abundant at the Loihi seamount hydrothermal vents and play a major role in Fe-oxide deposition. *App. Environ. Microbiol.* **68**, 3085-3093 (2002).
22. Templeton, A. S., Staudigel, H. & Tebo, B. M. Diverse Mn(II)-oxidizing bacteria isolated from submarine basalts at Loihi Seamount. *Geomicrobiol. J.* **22**, 127-139 (2005).
23. Campbell, B. J. & Cary, S. C. Abundance of reverse tricarboxylic acid cycle genes in free-living microorganisms at deep-sea hydrothermal vents. *Appl. Environ. Microbiol.* **70**, 6282-6289 (2004).

24. Kato, S., Nakawake, M., Ohkuma, M. & Yamagishi, A. Distribution and phylogenetic diversity of cbbM genes encoding RubisCO form II in a deep-sea hydrothermal field revealed by newly designed PCR primers. *Extremophiles* **16**, 277-83 (2012).
25. Futamata, H., Harayama, S. & Watanabe, K. Group-specific monitoring of phenol hydroxylase genes for a functional assessment of phenol-stimulated trichloroethylene bioremediation. *Appl. Environ. Microbiol.* **67**, 4671-4677 (2001).
26. Martínez-Lavanchy, P.M. *et al.* Microbial toluene removal in hypoxic model constructed Wetlands occurs predominantly via the Ring monooxygenation pathway. *Appl. Environ. Microbiol.* **81**, 6241-6252 (2015).
27. Lozupone, C. & Knight, R. UniFrac: a new phylogenetic method for comparing microbial communities. *Appl. Environ. Microbiol.* **71**, 8228-8235 (2005).
28. Pruesse, E., Peplies, J. & Glöckner, F. O. SINA: accurate high-throughput multiple sequence alignment of ribosomal RNA genes. *Bioinformatics* **28**, 1823-1829 (2012).
29. Barco, R. A. *et al.* New insight into microbial iron oxidation as revealed by the proteomic profile of an obligate iron-oxidizing chemolithoautotrophy. *Appl. Environ. Microbiol.* **81**, 5927-5937 (2015).
30. Jewell, T. N. M., Karaoz, U., Brodie, E. L., Williams, K. H. & Beller, H. R. Metatranscriptomic evidence of pervasive and diverse chemolithoautotrophy relevant to C, S, N and Fe cycling in a shallow alluvial aquifer. *ISME J.* **10**, 2106-2117 (2016).
31. Lopez-Gutierrez, J. C. *et al.* Quantification of a novel group of nitrate-reducing bacteria in the environment by real-time PCR. *J. Microbiol. Meth.* **57**, 399-407 (2004).
32. Hallin, S., Jones, C. M., Schloter, M. & Philippot, L. Relationship between N-cycling communities and ecosystem functioning in a 50-year-old fertilization experiment. *ISME J.* **3**, 597-605 (2009).
33. Hegler, F., Losekann-Behrens, T., Hanselmann, K., Behrens, S. & Kappler, A. Influence of seasonal and geochemical changes on the geomicrobiology of an iron carbonate mineral water spring. *Appl. Environ. Microbiol.* **78**, 7185-7196 (2012).
34. Coolen, M. J. *et al.* Putative ammonia-oxidizing Crenarchaeota in suboxic waters of the Black Sea: a basin-wide ecological study using 16S ribosomal and functional genes and membrane lipids. *Environ. Microbiol.* **9**, 1001-1016 (2007).
35. Pouliot, J., Galand, P. E., Lovejoy, C. & Vincent, W. F. Vertical structure of archaeal communities and the distribution of ammonia monooxygenase A gene variants in two meromictic High Arctic lakes. *Environ. Microbiol.* **11**, 687-699 (2009).
36. Ochsenreiter, T., Selezi, D., Quaiser, A., Bonch-Osmolovskaya, L. & Schleper, C. Diversity and abundance of *Crenarchaeota* in terrestrial habitats studied by 16S RNA surveys and real time PCR. *Environ. Microbiol.* **5**, 787-797 (2003).
37. Tourna, M. & Freitag, T. E. Growth, activity and temperature responses of ammonia-oxidizing archaea and bacteria in soil microcosms. *Environ. Microbiol.* **10**, 1357-1364 (2008).
